# Comparative cell biological study of in vitro antitumor and antimetastatic activity on melanoma cells of GnRH-III-containing conjugates modified with short-chain fatty acids

**DOI:** 10.3762/bjoc.14.226

**Published:** 2018-09-26

**Authors:** Eszter Lajkó, Sarah Spring, Rózsa Hegedüs, Beáta Biri-Kovács, Sven Ingebrandt, Gábor Mező, László Kőhidai

**Affiliations:** 1 Department Genetics, Cell- and Immunobiology, Semmelweis University, Nagyvárad tér 4., 1089 Budapest, Hungary; 2 Department of Informatics and Microsystem Technology, University of Applied Sciences Kaiserslautern, Amerikastraße 1, 66482 Zweibrücken, Germany; 3 Research Group of Peptide Chemistry, Hungarian Academy of Sciences, Eötvös Loránd University, Pázmány Péter sétány 1/A, 1117 Budapest, Hungary; 4 Eötvös Loránd University, Faculty of Science, Institute of Chemistry, Pázmány Péter sétány 1/A, 1117 Budapest, Hungary

**Keywords:** drug-targeting conjugates, gonadotropin-releasing hormone-III, holographic microscopy, impedimetry, short-chain fatty acids

## Abstract

**Background:** Peptide hormone-based targeted tumor therapy is an approved strategy to selectively block the tumor growth and spreading. The gonadotropin-releasing hormone receptors (GnRH-R) overexpressed on different tumors (e.g., melanoma) could be utilized for drug-targeting by application of a GnRH analog as a carrier to deliver a covalently linked chemotherapeutic drug directly to the tumor cells. In this study our aim was (i) to analyze the effects of GnRH-drug conjugates on melanoma cell proliferation, adhesion and migration, (ii) to study the mechanisms of tumor cell responses, and (iii) to compare the activities of conjugates with the free drug.

**Results:** In the tested conjugates, daunorubicin (Dau) was coupled to ^8^Lys of GnRH-III (GnRH-III(Dau=Aoa)) or its derivatives modified with ^4^Lys acylated with short-chain fatty acids (acetyl group in [^4^Lys(Ac)]-GnRH-III(Dau=Aoa) and butyryl group in [^4^Lys(Bu)]-GnRH-III(Dau=Aoa)). The uptake of conjugates by A2058 melanoma model cells proved to be time dependent. Impedance-based proliferation measurements with xCELLigence SP system showed that all conjugates elicited irreversible tumor growth inhibitory effects mediated via a phosphoinositide 3-kinase-dependent signaling. GnRH-III(Dau=Aoa) and [^4^Lys(Ac)]-GnRH-III(Dau=Aoa) were shown to be blockers of the cell cycle in the G2/M phase, while [^4^Lys(Bu)]-GnRH-III(Dau=Aoa) rather induced apoptosis. In short-term, the melanoma cell adhesion was significantly increased by all the tested conjugates. The modification of the GnRH-III in position 4 was accompanied by an increased cellular uptake, higher cytotoxic and cell adhesion inducer activity. By studying the cell movement of A2058 cells with a holographic microscope, it was found that the migratory behavior of melanoma cells was increased by [^4^Lys(Ac)]-GnRH-III(Dau=Aoa), while the GnRH-III(Dau=Aoa) and [^4^Lys(Bu)]-GnRH-III(Dau=Aoa) decreased this activity.

**Conclusion:** Internalization and cytotoxicity of the conjugates showed that GnRH-III peptides could guard Dau to melanoma cells and promote antitumor activity. [^4^Lys(Bu)]-GnRH-III(Dau=Aoa) possessing the butyryl side chain acting as a “second drug” proved to be the best candidate for targeted tumor therapy due to its cytotoxicity and immobilizing effect on tumor cell spreading. The applicability of impedimetry and holographic phase imaging for characterizing cancer cell behavior and effects of targeted chemotherapeutics with small structural differences (e.g., length of the side chain in ^4^Lys) was also clearly suggested.

## Introduction

The application of more selective, targeted drugs has become increasingly important in the treatment of tumors, where the use of chemotherapeutics with low therapeutic index is restricted by the adverse events coming from the toxicity of these drugs to normal cells [1]. One of the most promising approaches to diminish this kind of cytotoxic effects on healthy tissues is the employment of drug delivery systems directed specifically to cancer cells. The chemotherapeutic drug targeting is often based on the receptors for certain peptide hormones that are preferentially expressed by cancer cells. The utilization of these receptors for cancer cell targeting allows for minimizing the toxic side effects and producing high drug concentration selectively at the tumor site. In general, drug delivery systems consist of a targeting moiety in order to recognize a receptor on tumor cells and a cytotoxic drug covalently linked directly or through a suitable linker [1–2].

The gonadotropin-releasing hormone receptor (GnRH-R) is one of the receptors overexpressed on a wide range of tumors, and has limited expression in normal peripheral tissues [3]. The GnRH itself is a decapeptide hormone, which is responsible for the regulation of gonadal steroidogenesis and gametogenesis by integrating the nervous and endocrine system in the pituitary gland [4]. Regarding the targeted chemotherapy, it is highly advantageous that several native GnRH analogs, including the two human isoforms (GnRH-I and GnRH-II), and their synthetic derivatives have been reported to exert an antiproliferative effect in different types of tumors related (e.g., breast, endometrial cancer [5]) and unrelated (e.g., melanoma, colon carcinoma [6–7]) to reproductive organs. Considering these aspects, the GnRH could serve as a targeting unit with the aim to increase the concentration of an attached cytotoxic drug at the tumor cells overexpressing GnRH-R, and to decrease the unnecessary exposure of normal cells lacking GnRH-R [8]. Once the drug-targeting conjugate binds to its tumor-specific receptor, the receptor–conjugate complex can internalize into the cells by receptor-mediated manner, where the attached drug should be released from the conjugate in order to exert its antineoplastic activity [1].

The first GnRH-drug hybrids were developed by Schally and co-workers [9]. One of their most efficient conjugates was the zoptarelin doxorubicin (formerly known as AEZS-108 or AN-152), in which the superagonist [D-^6^Lys]-GnRH-I allows the tumor targeting of the traditional chemotherapeutical drug doxorubicin covalently linked via an ester bond [3,10]. However, while in the phase II trial, zoptarelin doxorubicin showed promising antitumor activity combined with the lower rate of adverse effects in recurrent endometrial cancers [11], in the phase III study, there was no meaningful difference between the patients treated with zoptarelin doxorubicin or doxorubicin with respect to efficacy of agents and incidence of adverse effects (e.g., cardiac disorders typical for anthracyclines) [12]. The adverse effects were supposed to be related to (i) doxorubicin released early from the conjugate because of the instability of the ester linkage and (ii) [D-^6^Lys]-GnRH-I induced endocrine side effects [8]. Therefore, recent strategies for the targeted chemotherapy favor conjugation methods resulting in a better stability of a conjugate in systemic circulation as well as GnRH derivatives with high affinity for tumor’s GnRH-R and negligible endocrine activity [13–14].

The GnRH-III (Glp-His-Trp-Ser-His-Asp-Trp-Lys-Pro-Gly-NH_2_), a native variant of GnRH, could display strong antiproliferative effects in the breast, prostate, colon carcinoma cell lines, whereas induce 500–1000 times less LH-release than GnRH-I derivatives [14]. In our previous studies, the side chain of Lys^8^ was used for attachment of daunorubicin (Dau) via a more stable oxime bond through an aminooxyacetyl (Aoa) linker to form different drug-containing conjugates [15–16]. Based on the enzymatic stability and capability of different Dau–GnRH-III conjugates to providing appropriate intracellular drug release [15], the oxime bond was used for coupling Dau to GnRH-III or its derivatives in the later studies.

There are some factors that could fundamentally limit the effectiveness of the GnRH-III-based targeting: (i) the relatively rapid proteolytic degradation of the peptide part [17], (ii) the variable density of the GnRH-R on cancer cells [18], (iii) the slow receptor-mediated endocytosis of the receptor–conjugate complex and (iv) the desensitization of GnRH-R [4,18].

The ^3^Trp-^4^Ser bond is a most susceptible site to be cleaved by proteolytic enzymes (e.g., chymotrypsin, angiotensin-converting enzyme). The substitution of Ser^4^ by its *N*-methyl analog (N-Me-Ser), or acetyl-Lys (Lys(Ac)) could improve the proteolytic stability of conjugates [17]. The results of in vitro and in vivo assays demonstrated that the conjugate containing Lys(Ac) in position 4 and Dau in position 8 ([^4^Lys(Ac)]-GnRH-III(Dau=Aoa)) had higher tumor growth inhibition activity than the unmodified GnRH-III-based one (GnRH-III(Dau=Aoa)) [17]. It is worth mentioning that the free Lys in this position also increased the in vitro cytostatic effect of the conjugate; however, its cellular uptake and enzyme stability were even lower than the parent conjugate had [17]. Therefore, it was not used in any further experiments. Based on these findings next generation of Dau–GnRH-III bioconjugates was developed in which Ser in position 4 was replaced with Lys acylated with short-chain fatty acids with 2–6 and 13 carbon atoms by Hegedüs et al. [19]. The replacement of ^4^Ser by Lys with a chain of 2–6 carbon atoms resulted in an increased cytostatic effect, while the conjugate with myristic acid (13 carbon atoms) had a lower activity compared to the GnRH-III(Dau=Aoa). Among these conjugates, [^4^Lys(Bu)]-GnRH-III(Dau=Aoa) containing butyric acid (Bu) acylated at the ^4^Lys residue was proved to be the most potent one in all different assays (enzymatic stability, cellular uptake, and in vitro antitumor activity) on HT-29 colon carcinoma cell lines [19]. The different hydrophobicity of conjugates having a short, fatty acid side chain (2–6 carbon atoms) could be excluded as an explanation for their different enzymatic stability and cellular uptake because these conjugates were found to have similar octanol–water partition index and membrane permeability. Nevertheless, the higher hydrophobicity (lower solubility) of the conjugate with myristic acid seemed to correlate with its increased stability, cellular uptake and weaker antitumor activity [17,19]. The increased cytostatic activity of Dau–GnRH-III conjugates acylated by a short-chain fatty acid as a “second drug” can be due to the known potential of short-chain fatty acids – especially butyric acid – to induce apoptosis in various tumor cell lines (e.g., colon [20], breast cancer cells [21]). Based on a former receptor binding experiment, the conjugates with Lys(Ac) or Lys(Bu) have a more suitable structure for receptor binding, which is even more preferential in case of the butyryl side chain; however, the even longer side chain linked to ^4^Lys could negatively affect the fitting of the conjugates to the N-terminal part of the GnRH-R. On the basis of these findings [^4^Lys(Bu)]-GnRH-III(Dau=Aoa), was chosen for the further studies (e.g., in vivo experiments [22]) to evaluate the suitability of this conjugate for targeted chemotherapy.

Malignant melanoma, despite the improving chemotherapeutic and surgical strategies, remains the leading cause of skin cancer deaths. The strong ability to disseminate metastases and to develop resistance to chemotherapy results in poor prognosis especially in advanced cases [23]. The expression GnRH-R was demonstrated in a very high percentage of human melanoma specimens derived from primary tumors or metastases and cell lines [24]. Activation of these receptors by means of GnRH agonists was shown to significantly decrease the proliferation and the motility of melanoma cell lines and the tumor growth inhibitory effect of a drug-containing GnRH conjugate (AN-207) clearly indicated that GnRH-R receptors are suitable for targeted tumor therapy [24,26]. Besides the well-established antitumor activity of GnRH variants (e.g., goserelin), their negative effects on tumor cell migration and invasion have been also demonstrated in melanoma cell lines [25–26].

There are also evidences that the short-chain fatty acids, including sodium butyrate and valproic acid, could inhibit the proliferation of melanoma cells both in in vitro (e.g., A2058 [27], B16 cell lines [28]) and in vivo experiments [28–29] or could abrogate the anticancer drug resistance as they are co-administrated with other chemotherapeutics [29–30]. Nevertheless, there is some controversy about the effects of short-chain fatty acids on the metastatic ability of melanoma cells, since both pro-invasive [31] and anti-invasive [29,32] activities have been described.

**Figure 1:**
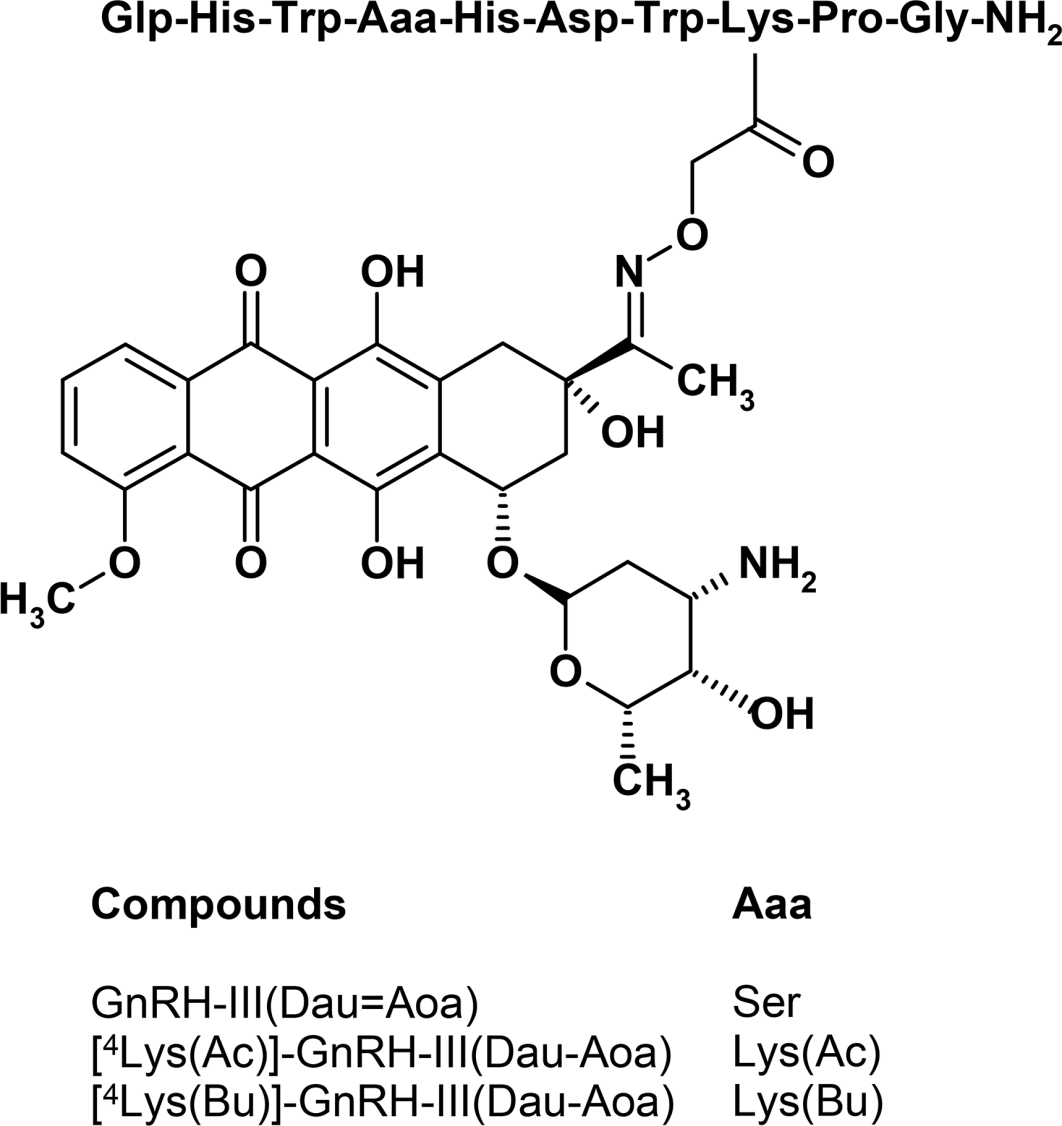
Schematic structure of Dau-conjugated GnRH-III or its derivatives containing ^4^Lys with acetyl or butyryl (Bu) side chain.

Taken together the aforementioned findings and considering the key roles of impaired adhesion and migratory phenotype of tumor cells in metastatic dissemination, we assumed that the Dau–GnRH-III conjugates substituted with short-chain fatty acids containing Lys may have not only the cytotoxic activity but also modulatory effects on cell adhesion and migration of melanoma cells. In our present work the effects of 3 Dau-GnRH-III conjugates – those carrying ^4^Lys with acetyl or butyryl side chain a “second drug” ([^4^Lys(Ac)]-GnRH-III(Dau=Aoa), [^4^Lys(Bu)]-GnRH-III(Dau=Aoa)) and the parent conjugate GnRH-III(Dau=Aoa) (Figure 1) – were investigated in respect of their cell biological activity and their applicability for targeted melanoma therapy. The significance of the above-described modification in position 4 of GnRH-III was evaluated by characterization of the cellular uptake, the antiproliferative/cytotoxic activities, the cell adhesion and migration modulator effects of conjugates and their ability to induce apoptosis or cell cycle arrest in A2058 melanoma cell line.

## Results and Discussion

### Synthesis of Dau–GnRH-III conjugates

The modified GnRH-III derivatives were prepared by solid phase peptide synthesis (SPPS) using Fmoc/*t-*Bu strategy with the orthogonal protecting scheme as described before [17,19] and presented in detail in Supporting Information File 1. In all cases, a Mtt (methyltrityl) protecting group was applied to block the side chain of Lys in position 8. For the development of acylated Lys in position 4, the side chain of it was protected either with Dde (1-(4,4-dimethyl-2,6-dioxocyclohex-1-ylidene)ethyl) or with ivDde ((1-(4,4-dimethyl-2,6-dioxocyclohex-1-ylidene)isovaleryl). The previous one can be removed easier with 2% hydrazine in DMF (2 × 15 min) while ivDde needs higher hydrazine concentration (4% in DMF) and longer treatment (12 × 5 min) for the complete removal of the protecting group. However, ivDde is more stable in circumstances (2% DBU, 2% piperidine in DMF) used for the Fmoc removal. To avoid the unwanted Dde removal during the synthesis ivDde was applied in this study. After acylation of the free amino group on the side chain of ^4^Lys using either acetic or butyric anhydride, the Mtt protecting group was detached. Though the application of bis-Boc-aminooxyacetic acid to incorporate the Aoa moiety provided better results (10–15% better yield according to the previous studies) than Boc-Aoa-OH because the overreaction of the sterically unhindered nitrogen in the case of the later one, here the Boc-Aoa-OH was used. After these on resin modifications of the peptide chain, the peptide derivatives were removed with a mixture of 95% TFA, 2.5% TIS, and 2.5% water (v/v/v) for 2.5 h at room temperature (rt) and then precipitated with ice-cold diethyl ether followed by purification on RP-HPLC. Daunorubicin was attached to the purified peptides via oxime linkage that was formed under slightly acidic conditions (0.2 M NH_4_OAc buffer at pH 5) at rt overnight. The reaction mixture was injected directly to RP-HPLC to separate the unreacted excess of Dau. Conjugates (Figure 1) were analyzed and identified by analytical HPLC-MS and ESIMS suggesting the right composition of the conjugates (Table 1 and Figures S1–S3 in Supporting Information File 2). The purity of the drug-containing conjugates was over 97% in all cases and untinged from free Dau, that can cause a significant influence on biological assays.

**Table 1:** Analytical characteristics of Dau–GnRH-III conjugates

compounds	RP-HPLC (C-4) *t*_R_[min]^a^	RP-HPLC (C-18) *t*_R_ [min]^b^	ESIMS *MW*_calcd_/*MW*_exp_ [g/mol]^c^

GnRH-III(Dau=Aoa)	21.37	21.37	1841.89/1841.85
[^4^Lys(Ac)]-GnRH-III(Dau=Aoa)	21.83	21.52	1925.02/1924.14
[^4^Lys(Bu)]-GnRH-III(Dau=Aoa)	22.42	22.05	1953.07/1952.97

^a^Column: Hichrom, Vydac 214TP5 C4 (300 Å, 5 µm, 250 × 4.6 mm) as a stationary phase. Linear gradient elution (0 min 0% B; 5 min 0% B; 40 min 90%). ^b^Column: Macherey-Nagel, Nucleosil C18 (100 Å, 5 µm, 250 × 4.6 mm) as a stationary phase. Linear gradient elution (0 min 0% B; 5 min 0% B; 30 min 90%). ^c^Bruker Daltonics Esquire 3000+ ion trap mass spectrometer. Dau: daunorubicin

### Cellular uptake of conjugates compared to Dau

An initial western blot study was done by using polyclonal anti-GnRH-R antibody (Proteintech Group, Rosemont, IL, USA) to detect the expression of GnRH-R in A2058 melanoma cells. Lysate of GnRH-R positive HT-29 colon carcinoma cells [33] was used as a positive control. Western blot analysis could reveal the presence of GnRH-R in A2058 cells; however, besides the band at approximately 37 kDa indicating the nascent full-length GnRH-R protein, there were bands in higher molecular weight in both samples (Figure S4 in Supporting Information File 2). The presence of extra bands could be explained by the different glycosylated variant of GnRH-R [34]. It is worth mentioning that based on this results it is hard to quantify or compare the amount of GnRH-R protein in these cell types.

**Figure 2:**
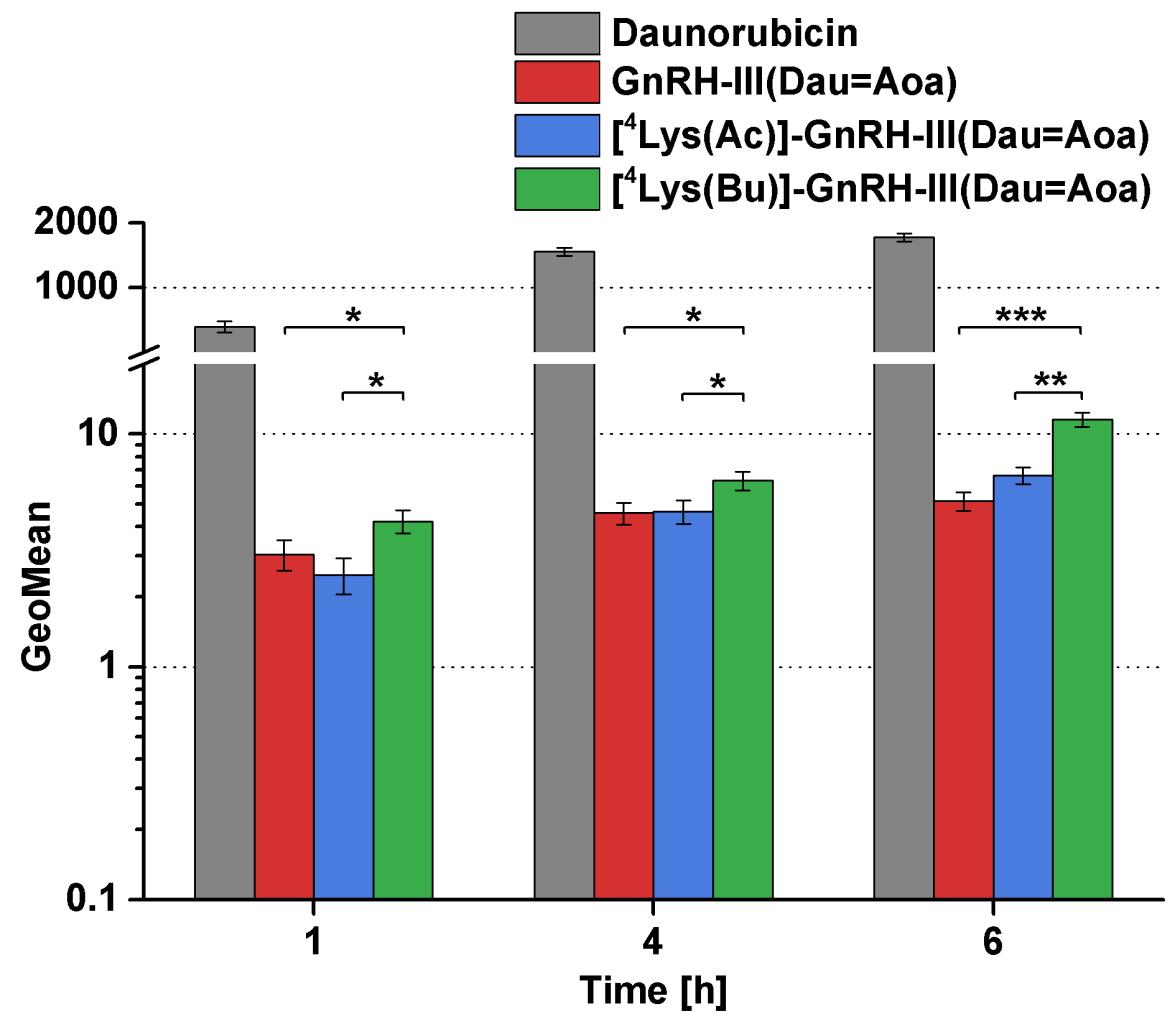
Cellular uptake of Dau–GnRH-III conjugates and free daunorubicin (Dau) by A2058 cells. Cellular uptake of the compounds was studied in 10^−5^ M concentration. GeoMean (geometric mean channel) value is a dimensionless value and refers to the relative fluorescence intensity. Data shown are mean of 2 parallels ± SD. The levels of significance are shown as follows: *: *p* < 0.05; **: *p* < 0.01, ***: *p* < 0.001.

For investigation and comparison of the cellular uptake of the conjugates, A2058 cells were treated with the conjugates at 10^−5^ M concentration for 1, 4 and 6 h. The fluorescence intensity of intracellular Dau built in the conjugates and as the free drug was determined by flow cytometry (FACSCalibur; Becton Dickinson, San Jose, CA, USA). GeoMean (geometric mean channel) values normalized to the control are shown in Figure 2.

The conjugates were internalized by A2058 cells in a time-dependent manner. In case of all conjugates, the cellular uptake could already be observed after 1 h of incubation. Comparing the conjugates, the butyrate containing conjugate ([^4^Lys(Bu)]-GnRH-III(Dau=Aoa)) was taken up most effectively, while there was no difference between the intracellular fluorescence intensity of GnRH-III(Dau=Aoa) and [^4^Lys(Ac)]-GnRH-III(Dau=Aoa). Dau served as a positive control in this experiment and showed a high level of intracellular fluorescence. Considering that Dau is a small molecule and can diffuse through the plasma membrane while the conjugates can enter the cells by receptor-mediated endocytosis with low capacity, this large-scale difference in the intracellular fluorescence intensity between the free Dau and the conjugates is not surprising. In addition, the free Dau has a ca. 10 times higher fluorescent intensity than the conjugates [35]. Comparing these results with the previous findings [19], [^4^Lys(Bu)]-GnRH-III(Dau=Aoa) was shown to be the best-internalized conjugate and this ability proved to be independent of the tumor cells.

### Antiproliferative/cytotoxic effect of conjugates

One of the major requirements for a drug-delivery conjugate is the ability to provide the antitumor activity of the attached drug inside the cells. The antiproliferative/cytotoxic effect of conjugates was investigated by an impedimetric technique, xCELLigence System (ACEA Biosciences, San Diego, CA, USA). The real-time measurement of the impedance change, which is in direct correlation with the number of adhered cells on an electrode surface, makes this impedimetric assay sensitive enough for cytotoxicity experiments [36]. In the event of a cytotoxic compound, the cells detach from the electrode surface and a drop in the impedance – given as Cell index values – could be observed.

According to the time-course study, the conjugates elicited their tumor-growth inhibitory effect only at high concentrations (10^−5^ to 10^–4^ M) and in long-term manner; 15–20 h after the treatment the Cell index values constantly decreased, which means that the cell viability was gradually lower as the time passed. Dau had a more immediate effect (0–5 h) in 10^–6^ to 10^−4^ M range (Figure S5 in Supporting Information File 2). IC_50_ values – a concentration that decreases the cell viability by 50% – were calculated from Cell index values obtained at 48 h and 72 h for each concentration and used for comparing the effects of conjugates. It is clearly seen that the presence of acylated Lys could increase almost 10-fold the antitumor activity (*p* < 0.001) of parent conjugate (GnRH-III(Dau=Aoa)). In case of the acylated ^4^Lys-containing conjugates, [^4^Lys(Bu)]-GnRH-III(Dau=Aoa) had a slightly but not significantly higher cytotoxic activity than that of [^4^Lys(Ac)]-GnRH-III(Dau=Aoa) after 48 h or 72 h of incubation (Table 2).

**Table 2:** Determination of the long-term cytotoxic effect of GnRH-III-based conjugates and daunorubicin (Dau).

compounds	48 h IC_50_ (μM)	72 h IC_50_ (μM)

daunorubicin	0.19 ± 0.017	0.053 ± 0.009
GnRH-III(Dau=Aoa)	42.6 ± 6.54	14.8 ± 1.47
[^4^Lys(Ac)]-GnRH-III(Dau=Aoa)	6.10 ± 0.59	4.94 ± 0.45
[^4^Lys(Bu)]-GnRH-III(Dau=Aoa)	5.89 ± 0.91	4.19 ± 0.31

IC_50_ values were calculated by fitting a sigmoidal dose-response curve with RTCA 2.0 software. Data shown represent the mean ± SD of three parallel measurements.

A similar enhanced antitumor activity was also detected for conjugates modified with acylated ^4^Lys in the HT-29 human colon [19], LNCaP [17] and DU145 [37] human prostate cancer cell lines compared to the conjugate containing native GnRH-III sequence. Despite the increased activity of conjugates substituted with acylated ^4^Lys, they displayed their dose-dependent cytotoxic activity at higher concentrations comparing to the free Dau. This difference could be explained by the internalization ability of the compounds. The action of conjugates requires their receptor-mediated internalization and the lysosomal degradation, while Dau could diffuse through the plasma membrane and exerts its antitumor activity by intercalating directly to DNA. Furthermore, it was previously demonstrated that the smallest Dau-containing fragment (H-Lys(Dau=)-OH)) formed via lysosomal degradation of this type of conjugates had a lower binding affinity to DNA, than the free drug [35]. The two-fold higher intracellular fluorescence intensity of Dau was accompanied by almost two-fold higher cytotoxic activity compared to the conjugates. By comparing the effects of the parent conjugate and the acylated ^4^Lys-containing ones, there was no clear correlation between their cellular uptake and long-term cytotoxicity. To interpret the significance of the modification with acylated Lys it would be useful to confront the effects of the acetyl-Lys-containing conjugates with the non-acylated Lys-containing one ([^4^Lys]-GnRH-III(Dau=Aoa)). However, the substitution with ^4^Lys was formerly shown to lead increased cytostatic activity on different cancer cell lines (e.g., MCF-7, HT-29), this conjugate was less stable against different digestive enzymes and was taken up less effectively compared to GnRH-III(Dau=Aoa) [17]. Therefore, ([^4^Lys]-GnRH-III(Dau=Aoa) was not involved in our present study.

**Figure 3:**
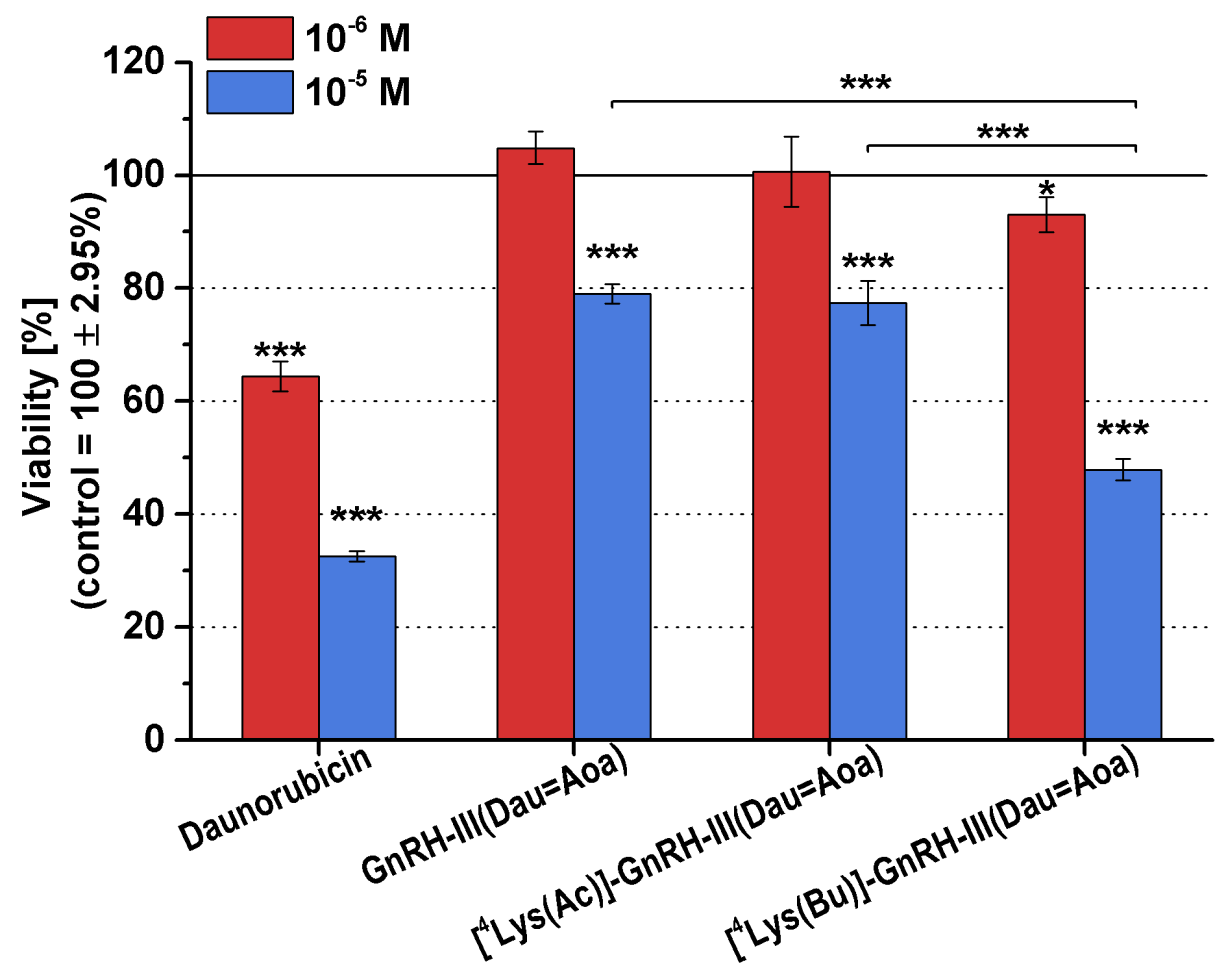
Short-term growth inhibitory effects of the conjugates and daunorubicin (Dau) on A2058 cells*.* The model cells were incubated with the compounds at 10^−6^ and 10^−5^ M concentrations for 6 h. The ‘Viability’ is expressed as a percentage of the control. Data shown in the figure represent mathematical averages of six parallels and ± S.D. values. The level of significance is shown as follows: *: *p* < 0.05; ***: *p* < 0.001.

The effect of the short-term treatment (6 h) with the conjugates and Dau was also determined. The viability of A2058 cells treated with the 10^−6^ and 10^−5^ M compounds was detected by alamarBlue^®^-assay after washing out the substances from the cells and a further 48 h of culturing. The results of the short-term growth inhibitory effect are presented in Figure 3.

Compared to the free drug, all conjugates elicited lower growth inhibitory effects. The conjugate containing a butyryl side chain ([^4^Lys(Bu)]-GnRH-III(Dau=Aoa)) decreased slightly, but significantly the cell viability already at a concentration of 10^−6^ M, while it had a strong antiproliferative/cytotoxic effect at 10^−5^ M. [^4^Lys(Ac)]-GnRH-III(Dau=Aoa) and GnRH-III(Dau=Aoa) displayed a similar growth inhibitory effect, respectively, but only at 10^−5^ M concentration, and they proved to be significantly less effective than [^4^Lys(Bu)]-GnRH-III(Dau=Aoa).

However, the tumor growth inhibitory effect of the long-term treatment manifested after 17–24 h, the short-term exposure (6 h) could still cause a significant antitumor effect, suggesting that the conjugates could irreversibly reduce the melanoma cell viability. In contrast to the long-term treatments, the order of the internalization rate and the antitumor activity of the compounds was the same: Dau >> [^4^Lys(Bu)]-GnRH-III(Dau=Aoa) > [^4^Lys(Ac)]-GnRH-III(Dau=Aoa) ~ GnRH-III(Dau=Aoa). While there were no significant differences between [^4^Lys(Ac)]-GnRH-III(Dau=Aoa) and [^4^Lys(Bu)]-GnRH-III(Dau=Aoa) in terms of long-term (48 h) toxicity, the conjugate with butyryl side chain proved to be more effective after 6 h-long exposure. It seems that the short-term effects of conjugates could be due to their different internalization kinetics, or different mechanism of their action (e.g., induction of apoptosis or inhibition of cell cycle) rather than in case of the long-term activities. This could mean that once the acylated conjugates are internalized into the cell and completely degraded in the lysosome they could behave not so differently with each other in a long-term manner.

It is important to emphasize that all of the conjugates were stable in human serum for at least 24 h [17,19,35]. Therefore, in case of the in vitro conditions (the serum content of the medium was 10%), it could be excluded that the different stability of conjugates and the premature release of Dau would cause the difference in their time-dependent antitumor activities. It was previously shown that all Dau–GnRH-III conjugates were degraded already after short-term incubation (2–8 h) with lysosomal homogenate, leading to the formation of various peptide fragments and Dau-containing metabolites such as H-Lys(Dau=Aoa)-OH [17,19,35]. LC–MS spectra recorded during the lysosomal degradation studies could also indicate the cleavage of an amide bond between the side chain of ^4^Lys and the fatty acids [19]. This result suggests that besides the Dau-metabolites the fatty acids could also be released and – as second drugs – could contribute to the tumor growth inhibitory action of conjugates. It is assumed that the higher short-term cytotoxic activity of [^4^Lys(Bu)]-GnRH-III(Dau=Aoa) could be attributed to the presence of butyrate, which has been demonstrated to inhibit the proliferation as well as to induce apoptosis and cell cycle arrest in different cell lines (e.g., colon carcinoma, melanoma, T-cell lymphoma) as a histone deacetylase inhibitor and/or via activation of orphan G-protein-coupled receptor GPR43 [20,27,38]. In short-term, the butyryl side chain of the intact conjugate may exert its tumor suppressor effect via acting on cell surface receptor (e.g., GPR43) [20] or after the conjugate being internalized, the released butyrate may induce apoptosis via caspase-3 [39]. It is possible that the Dau-metabolites formed inside the cells need longer time to exert their more prominent antitumor effect, which could explain why the long-term cytotoxic effect was almost the same for the two acylated conjugates.

It has been reported that doxorubicin (an analog of Dau) had a synergistic effect with histone deacetylase inhibitors (e.g., prodrug of butyric acid) in several malignant cell lines [38]. This finding could also be a possible explanation for the increased cytotoxic effect of the conjugates containing acylated Lys compared to the parent one.

### Involvement of phosphatidylinositol 3-kinase in the antitumor effects of conjugates

Binding of GnRH or its conjugates to the GnRH-R receptor on tumor cells could stimulate different signaling elements (e.g., phosphatidylinositol 3-kinase – PI3K, mitogen-activated protein kinases) and effector proteins, which could play a significant role in the antitumor activity of a drug-containing conjugate [40].

The association of PI3K activation with the antitumor activity of the conjugates was determined by pretreating the cells with PI3K inhibitors (wortmannin – W and LY294002 – LY). The antitumor effect of the conjugates and Dau on the cells pretreated with PI3K inhibitors or with DMSO (solvent of the inhibitors) was assessed by an alamarBlue-assay after 48 h of incubation. The results of the PI3K-assay were given by calculating the inhibition index as the ratio of the viability of the pretreated and control (DMSO-treated) cell populations being incubated with medium or conjugates (10^−5^ M). The smaller inhibition index is than 100%, the lower antitumor activity compounds elicited on the cells pretreated with PI3K inhibitors than the control cells.

According to the inhibition indices shown in Table 3, none of the inhibitors had any influence on the antitumor activity of Dau and GnRH-III(Dau=Aoa). On the contrary, the antitumor effect of conjugates modified in position 4 was sensitive to the inhibitors. The effect of [^4^Lys(Bu)]-GnRH-III(Dau=Aoa) could be slightly reduced by both inhibitors (W: 91.4%, LY: 80.0%), while in case of [^4^Lys(Ac)]-GnRH-III(Dau=Aoa), only the pretreatment with W resulted in this kind of inhibition (89.3%) (Table 3). These results indicated that the conjugates containing acylated Lys in position 4 exerted their cytotoxic effect, at least in part, via a PI3K-dependent mechanism, while the PI3K seemed to be not involved in the cytotoxicity of the parent conjugate (GnRH-III(Dau=Aoa)). These results are in good agreement with previous studies about the involvement of PI3K signaling pathway in the pro-apoptotic effects of GnRH analogs on prostate [41] and ovarian [42] cancer cells as well as in the chemotaxis of leukemic cells [43] induced by GnRH-III derivatives.

**Table 3:** The effect of the inhibition of phosphatidylinositol 3-kinase on the antitumor activity of conjugates and daunorubicin (Dau).

compounds	inhibition index^a^ [%]	

	wortmannin	LY294002

daunorubicin	121.0 ± 8.1	100.7 ± 4.0
GnRH-III(Dau=Aoa)	102.6 ± 10.1	102.0 ± 3.9
[^4^Lys(Ac)]-GnRH-III(Dau=Aoa)	89.3 ± 5.4*	94.3 ± 4.3
[^4^Lys(Bu)]-GnRH-III(Dau=Aoa)	91.4 ± 2.0*	80.0 ± 9.1*

^a^The antitumor activity of the cells pretreated with wortmannin or LY294002 was characterized by inhibition index = (*G*_nh_ × *C*_c_)/(*C*_inh_ × *G*_c_) × 100%. Cells pretreated with DMSO were incubated with control medium (*C*_c_) or a compound (*G*_c_); cells pretreated with PI3K blockers were assayed for the control medium (*C*_inh_) or a compound (*G*_inh_). Data shown in the table were calculated from the averages of 6 parallels. The level of significance is shown as follows: *: *p* < 0.05.

### Apoptotic and cell cycle blocking effects of conjugates

In order to understand the mechanism of the antitumor effect of conjugates and consequently explain the difference in their activity, we determined whether these conjugates could induce apoptosis and/or cell cycle arrest in melanoma cells.

The pro-apoptotic effects of conjugates and Dau at 10^−5^ M concentration was studied after 24 h incubation by flow cytometry using FITC-annexin V (Sony Biotechnology, Weybridge, UK) and a novel image cytometer (NucleoCounter^®^ NC-250^TM^, ChemoMetec A/S, Lillerød, Denmark) using Vitabright-48™ (ChemoMetec A/S, Lillerød, Denmark), a cell permeable dye that reacts with thiol groups to form a fluorescent product. An inverse correlation has been shown between the concentration of thiols and progression of apoptosis; the level of thiols, and hence the fluorescence intensity of this dye decrease in response to induction of apoptosis [44].

Based on the percentages of apoptotic cells shown in Table 4, there was a good match between the values provided by these two different methods. As expected, the maximum apoptotic effect was detected in the Dau-treated group. Among the conjugates, only [^4^Lys(Bu)]-GnRH-III(Dau=Aoa) had a slight, but significant apoptotic activity compared to the control. In the case of both assays, GnRH-III(Dau=Aoa) and [^4^Lys(Ac)]-GnRH-III(Dau=Aoa) failed to exert any significant apoptotic effect in melanoma cells (Table 4).

**Table 4:** Apoptosis inducer effects of the conjugates and daunorubicin (Dau) in melanoma cells.

compounds	the ratio of apoptotic cells [%]	

	annexin V	Vitabright-48™

control	10.6 ± 0.51	8.7 ± 0.72
daunorubicin	33.6 ± 5.51***	30.9 ± 0.68***
GnRH-III(Dau=Aoa)	16.2 ± 3.28	10.6 ± 0.91
[^4^Lys(Ac)]-GnRH-III(Dau=Aoa)	13.4 ± 0.54	13.5 ± 0.91
[^4^Lys(Bu)]-GnRH-III(Dau=Aoa)	17.1 ± 1.83*	15.3 ± 0.89*

The model cell was incubated with the compounds at 10^−5^ M concentration for 24 h. The ‘ratio of apoptotic cells’ is expressed as a percentage of viable cells measured by flow cytometry or NucleoCounter^®^ NC-250^TM^. Data shown represent mathematical averages of two parallels and ± S.D. values. The level of significance is given as follows: *: *p* < 0.07; ***: *p* < 0.001.

**Figure 4:**
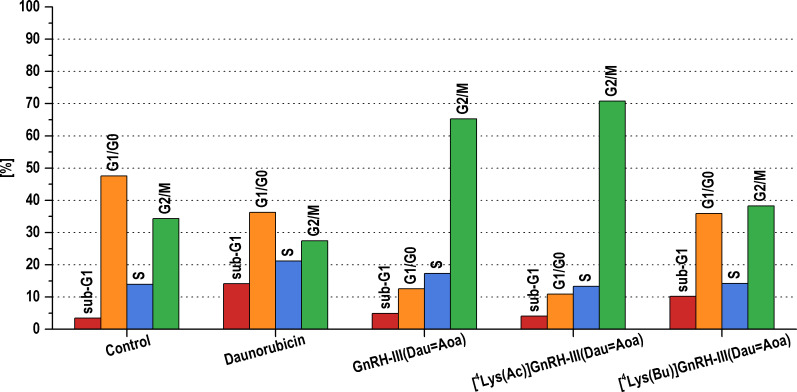
Effects of conjugates and daunorubicin (Dau) on cell cycle progression of A2058 melanoma cells. The model cell was incubated with the compounds at 10^−5^ M concentration for 24 h. Results are expressed as a percentage of cells in the sub-G1 area and the major phases (G1/G0, S and G2/M) of the cell cycle. Data shown in the figure represent mathematical averages of two parallel measurements.

Since the conjugates had no or minor apoptotic effect, the cell cycle kinetics of the cells treated with GnRH-III-based conjugates was also investigated to reveal the mechanism of growth inhibition of these conjugates. The distribution of the control and treated cells in the different cell cycle phases was analyzed by measurements of relative DNA contents of individual cells by flow cytometry after propidium iodide (Sigma-Aldrich, St. Louis, MO, USA) staining. The effects of conjugates and Dau on cell cycle phase distribution of A2058 cells are shown in Figure 4. The apoptotic inducer activity of Dau was also manifested in the pattern of cell cycle phases; the percentage of sub-G1 phase representing apoptotic cells (cell fragments) was increased after cells were treated with 10^−5^ M Dau up to 14.1% compared with 3.4% of the control. In parallel, a decrease in the proportions of cells in G1/G0 and G2/M phase and an increase in the percentage of S phase cells were also observed. Treatment with [^4^Lys(Bu)]-GnRH-III(Dau=Aoa) also resulted in the accumulation of cells in the sub-G1 phase to 10.2%, accompanied by a decrease in the percentage of cells in G1/G0 phase. Whereas, [^4^Lys(Ac)]-GnRH-III(Dau=Aoa) had a very similar effect on cell cycle progression as GnRH-III(Dau=Aoa). Cells treated with these conjugates showed higher G2/M populations (70.8% and 65.3%, respectively) and concomitant lower G1/G0 populations (10.9% and 12.6%, respectively) compared with the control (G2/M: 34.3% and G1/G0: 47.6%).

Overall, these results indicated that (i) the accumulation of the apoptotic population of melanoma cells might be partly responsible for the [^4^Lys(Bu)]-GnRH-III(Dau=Aoa)-induced inhibition of cell growth, while [^4^Lys(Ac)]-GnRH-III(Dau=Aoa) and GnRH-III(Dau=Aoa) could mediate their effect on melanoma cell proliferation via blocking cell cycle progression in G2/M phase. In a previous study, Pályi and his co-workers demonstrated that a conjugate containing GnRH analog + copolymer could also cause the accumulation of endometrial cancer cells in the G2/M phase [45]. In contrast to their [45] and our findings, different GnRH analogs were shown to inhibit the transition of G1 to S phase [46–47]. These studies also suggested that the greater tumor growth inhibitory effect of peptide conjugates than the free GnRH peptide could be explained by their different effects on cell cycle phase distribution [45,47]. Based on the findings about the different mechanism of [^4^Lys(Bu)]-GnRH-III(Dau=Aoa) than the other conjugates, it is assumed that its apoptotic effect could attribute to the presence of butyryl side chain, known for its ability to activate apoptosis [20,39].

### Cell adhesion modulator effect of the conjugates

**Figure 5:**
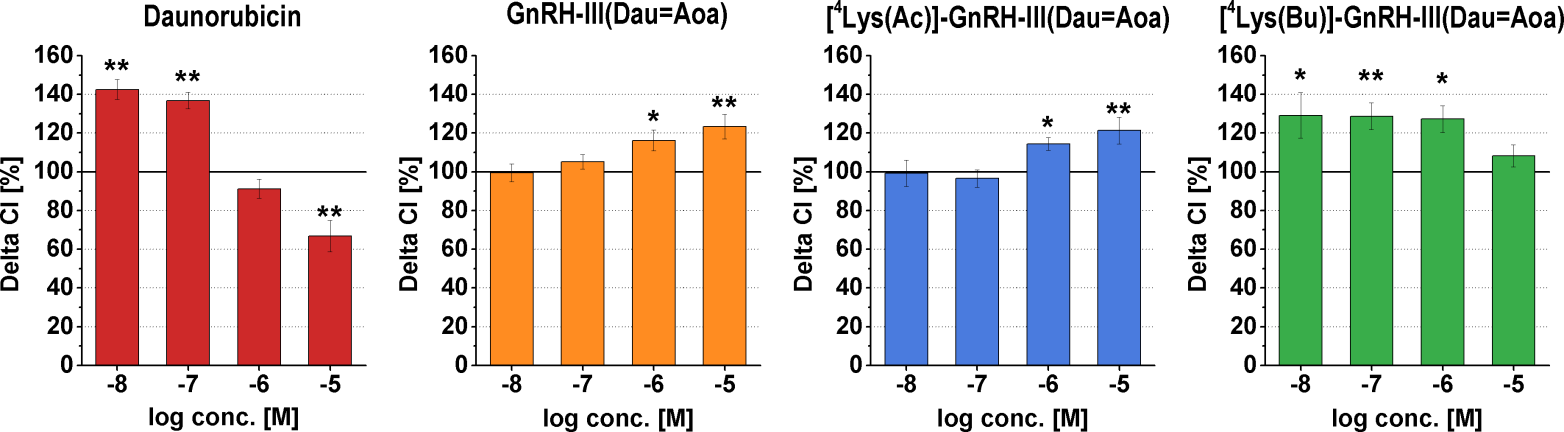
Effects of the conjugates and daunorubicin (Dau) on melanoma cell adhesion. The Delta CI (Delta Cell index) normalized to the control (control = 100%) refers to the difference of the Cell index value at the point in time of cell inoculation and the Cell index value at 3 h later. Data shown in the figures represent mathematical averages of three parallels and ± SD values. The levels of significance are shown as follows: *: *p* < 0.05; **: *p* < 0.01.

The dissemination of tumor cells is an important aspect of the tumor progression and could significantly affect the success of the targeted tumor therapy, as well. The first crucial steps in metastasis cascade are the impaired adhesion contacts and in parallel the increased motility of tumor cells. These two events substantially provide for tumor cells to detach from the primary tumor and migrate to the surrounding tissue [48–49]. Besides the tumor-selective antiproliferative/cytotoxic activity of the conjugates, they could be also desired to have an ability to interfere with the dissemination of cancer cells by modulating their adhesion and migration. In order to evaluate the conjugates as antimetastatic therapeutics, their effects on melanoma cell adhesion were investigated by the impedance-based xCELLigence System. The cell adhesion modulator effects of compounds were characterized by Delta Cell index values. These values were calculated for the rapid adhesion phase of A2058 – for the 3 hour long time interval after the cell seeding and treatment – and displayed in Figure 5.

Dau itself was able to induce the adhesion of A2058 cells at low concentrations (10^−8^ to 10^−7^ M), whereas a decrease was detected at 10^−5^ M, which could be explained by its immediate cytotoxic effect described above. In case of the adhesion measurements, GnRH-III(Dau=Aoa) and [^4^Lys(Ac)]-GnRH-III(Dau=Aoa) had very similar adhesion inducer effects at 10^−6^ to 10^−5^ M concentrations. The adhesion of A2058 cells was also increased by [^4^Lys(Bu)]-GnRH-III(Dau=Aoa), but at lower concentrations (10^−8^ to 10^−6^ M, Figure 5).

One of the first events of tumor progression is a decrease in anchorage dependency of tumor cells, which lead to their detachment and acquisition of migratory activity. One of the possible strategies to limit the local invasion of malignant cells is to increase their adhesion and consequently restrict their motility within the primary lesion [48]. Based on the detected adhesion inducer activity of Dau–GnRH-III conjugates, they might be effective in the prevention of tumor cell dissemination.

Holographic microscopic measurements were also performed to visualize the morphological changes induced by the conjugates and Dau. This novel technique provided several morphological parameters (e.g., surface area, optical thickness, eccentricity etc.) that allowed understanding of the results of impedance-based adhesion measurement. Holographic images were taken before and after the 3-hour treatment of A2058 cells with the compounds. The change in the morphological parameters shown in Table S1 in Supporting Information File 2 was calculated from a fold change during 3 h long treatment and this value was normalized to that of the control cells. The adhesion inducer effects of Dau and GnRH-III(Dau=Aoa) detected by the impedimetric assay were reflected in the morphometry analysis. Both compounds could increase the surface area of adhered melanoma cells (124.1% ± 4.64, *p* < 0.001 and 115.7% ± 4.67, *p* < 0.05), while the optical thickness was reduced but only in case of the GnRH-III(Dau=Aoa) treated cells (85.6 ± 1.45, *p* < 0.001). Although [^4^Lys(Ac)]-GnRH-III(Dau=Aoa) proved to be an adhesion inducer, but it had a negative or no effect on these morphological indices (surface area: 84.2 ± 3.32 – 92.5 ± 3.5; thickness: 95.7 ± 1.94 – 103.1 ± 2.21). Opposite tendencies were shown for [^4^Lys(Bu)]-GnRH-III(Dau=Aoa), it could increase slightly, but significantly the surface area (114.0 ± 5.64, *p* < 0.05) and in parallel reduce the optical thickness (85.7 ± 1.85, *p* < 0.01) at 10^−6^ M concentration.

This apparent lack of consistence between the results of impedimetry and the basic morphometric parameters provided by holographic microscopy can be attributed to some method- and cell-related factors. The change in impedance (Cell index) during cell adhesion depends on (i) the cell–cell junctions, (ii) the seal resistance related to the cleft height between the cell and the electrode surface as well as (iii) the membrane capacitance correlated well with the amount of attached membrane area. The stronger adhesion could be due to a decrease in the cleft height, without any change in the surface area of the attached cells [50]. This can be a possible explanation for the neutral effect of [^4^Lys(Ac)]-GnRH-III(Dau=Aoa) on the optical thickness and surface area at 10^−6^ to 10^−5^ M concentrations. The cellular shape could also influence the impedimetric results. The complex morphological parameters (e.g., eccentricity, irregularity and hull convexity) describing the shape or the circumference of the cells proved to be sensitive to the effects of the conjugates. The eccentricity and/or irregularity were elevated by GnRH-III(Dau=Aoa) (eccentricity: 107.4% ± 1.87, *p* < 0.05; irregularity: 110.9% ± 2.43, *p* < 0.05) and [^4^Lys(Bu)]-GnRH-III(Dau=Aoa) (irregularity: 117.8% ± 2.99, *p* < 0.01). Some holographic microscope-related factors could also influence the interpretation of the results. For example, the background noise could limit the vertical and lateral resolution of the instrument and consequently, the very thin parts of cells or cell spreading cannot be sensed perfectly [51].

### Effect of the conjugates on melanoma cell movement

Besides the altered tumor cell adhesion, there is growing evidence implicating tumor cell motility in early tumor invasion, and therapeutic targeting the migratory behavior of tumor cells within the primary tumor could limit local invasion [48]. To check the hypothesis that GnRH-based conjugates could inhibit the melanoma cell movement, their chemotactic (inducing vectorial migration) and chemokinetic (modulating locomotion) activities were investigated.

**Figure 6:**
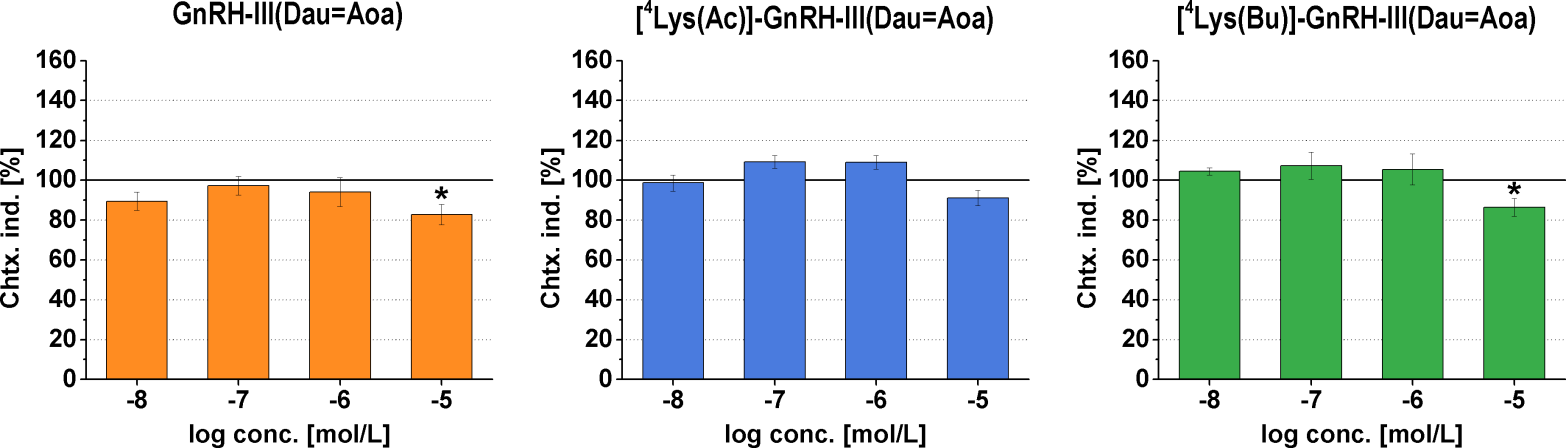
Chemotactic effects of Dau–GnRH-III conjugates on A2058 cell line. The ‘Chemotaxis index’ (Chtx. ind.) is expressed as a percentage of the control. Dau: daunorubicin. Data shown in the figures represent averages calculated for 8 parallels ± SD values. The level of significance is shown as *: *p* < 0.05.

Firstly the chemotaxis of A2058 cells towards the conjugates was measured by a NeuroProbe^®^ chemotaxis chamber. Both GnRH-III(Dau=Aoa) and [^4^Lys(Bu)]-GnRH-III(Dau=Aoa) proved to have weak, but significant chemorepellence in 10^−5^ M concentration (Figure 6), while the conjugate with ^4^Lys(Ac) elicited a rather neutral effect in the tested concentration range (10^−8^ to 10^−5^ M, Figure 6).

Considering the short-term treatments (0–3 h) in case of the measurements of cell movement and adhesion, the effects of conjugates on melanoma cell adhesion and movement are supposed to be mediated via GnRH-R activated signaling rather than via intracellular mechanisms induced after the internalization of conjugates. Although the conjugates showed an opposite activity on melanoma adhesion (increasing effects) and chemotaxis (decreasing or neutral effects), based on our previous studies about the receptor binding affinity of conjugates [19] and the chemotactic and adhesion modulator effect of native GnRH isoforms [19] it is assumed that the tested conjugates act as agonists on GnRH-R, but depending on the cellular function the resultant effects are different. It has been already reported for several tumor cell types, that depending on the cellular milieu or function, the GnRH analogs could elicit different – even opposite – actions [6,40]. For example, Aguilar-Rojas and his co-workers reported a similar combination of actions (invasion inhibitory and adhesion increasing effects) of a GnRH agonist in a breast cancer cell line [52]. It is also important to note that the effects on cell migration/chemotaxis and cell attachment are not independent cellular functions from each other. The cell-surface attachments basically influence the cell movement; the increased adhesiveness could result in a reduced cellular movement because of difficulty in realizing adhesion contacts to the substrate [53]. Our present results about the chemorepellent character of the conjugates appeared to be well-correlated to their effect on cell adhesion and cellular morphology of A2058 cells.

Next, the chemokinetic activities (inducing random cell movement or locomotion) of conjugates were investigated by monitoring the locomotion of A2058 cells with holographic microscopy under the condition that the conjugates (10^−7^ to 10^−5^ M) were added directly to the cells in a uniform concentration. For the characterization of cellular movement, three parameters – migration (shortest distance), motility (actual path) and motility speed (ratio of actual path and time) were quantified by tracking single cells in time-lapse videos recorded by a HoloMonitor^TM^ M4 microscope (Phase Holographic Imaging AB, Lund, Schweden).

**Figure 7:**
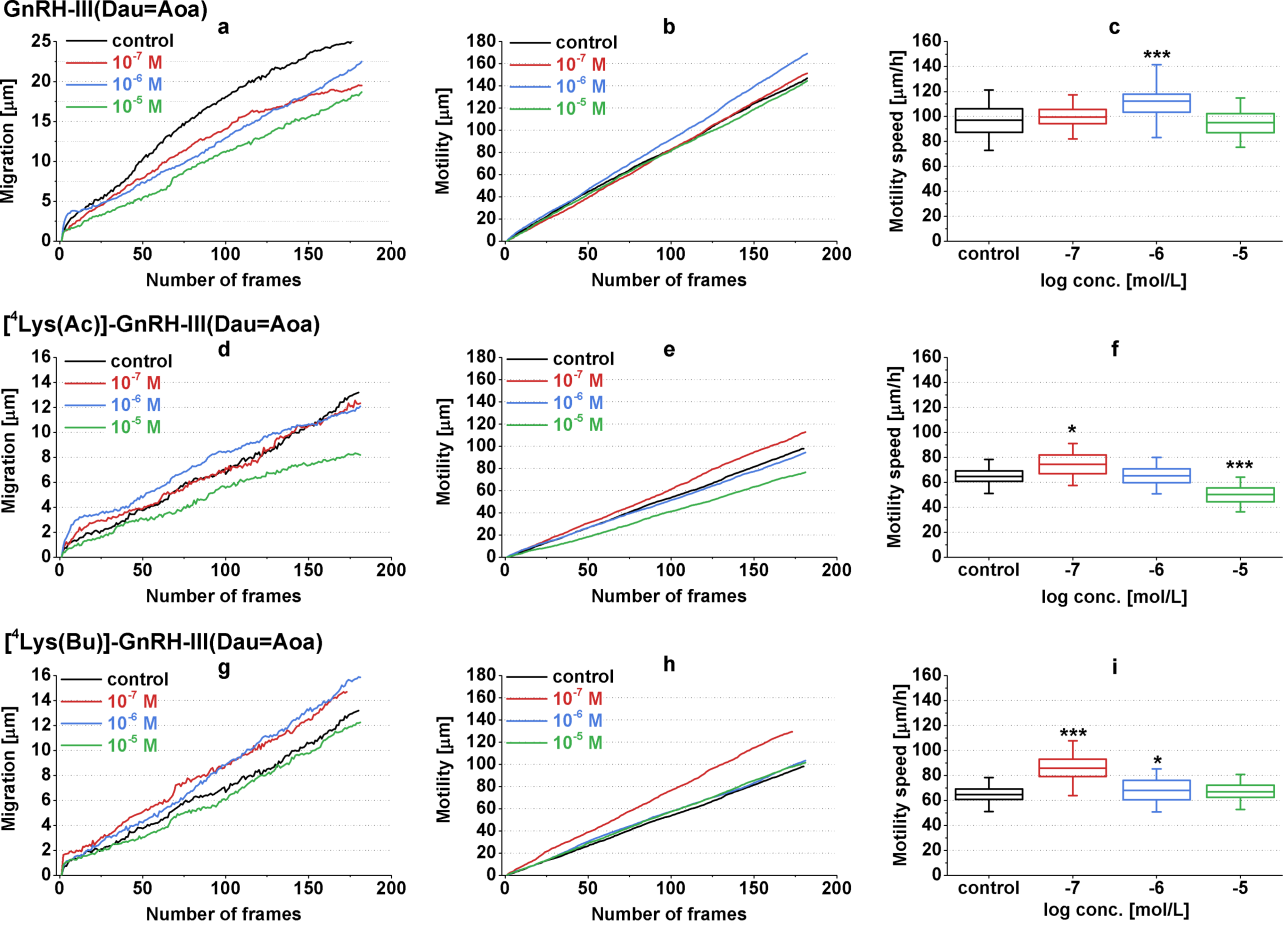
Effects of GnRH-III(Dau=Aoa) (a–c), [^4^Lys(Ac)]-GnRH-III(Dau=Aoa) (d–f) and [^4^Lys(Bu)]-GnRH-III(Dau=Aoa) (g–i) on the locomotion of A2058 cell line. The migration (a, d, g), motility (b, e, h) and motility speed (c, f, i) were investigated by HoloMonitor^TM^ M4 holographic microscopy. Dau: daunorubicin. Data shown in the figures represent averages calculated for 50 cell/group in 180 consecutive frames. The levels of significance are shown as follows *: *p* < 0.05; ***: *p* < 0.001.

Based on the results, the GnRH-III(Dau=Aoa) and the ^4^Lys(Bu)-containing derivative conjugates displayed rather negative effects on the melanoma cell locomotion (Figure 7). By comparing the results of GnRH-III(Dau=Aoa) to the control this conjugate decreased the migration of A2058 cells in a concentration-dependent manner (Figure 7a), while a slight increase in the motility and the motility speed could be detected but only at 10^−6^ M concentration (Figure 7b and c). The highest concentration of [^4^Lys(Bu)]-GnRH-III(Dau=Aoa) decreased the migration, the motility and the motility speed of A2058 cells compared to that of the control cells (Figure 7g–i). However, the motility was slightly increased by 10^−7^ M [^4^Lys(Bu)]-GnRH-III(Dau=Aoa), the migration was similar to the control group (Figure 7g,h). These results could indicate that GnRH-III(Dau=Aoa) and [^4^Lys(Bu)]-GnRH-III(Dau=Aoa) would rather keep the cells in place. On the contrary, [^4^Lys(Ac)]-GnRH-III(Dau=Aoa) could increase all of the parameters at 10^−7^ M concentration (Figure 7d–f), which means the cells travel further in a more winding path with a higher velocity than the control cells. The migration inducer effect of 10^−6^ M [^4^Lys(Ac)]-GnRH-III(Dau=Aoa) indicated a more directed movement of cells (Figure 7d).

In some cases (e.g., [^4^Lys(Ac)]-GnRH-III(Dau=Aoa)) concentration-dependent dual effects were observed. This kind of concentration dependence of GnRH effects is not unique in the literature. A similar diverse migratory response was found to GnRH-I actions in case of ovarian cell lines. This kind of biphasic effect could be explained by the presence of different GnRH receptors or depending on the concentration of a GnRH derivative/conjugate it could stimulate a different signaling pathway via a GnRH-R [40]. The opposite chemokinetic effects of the conjugates containing ^4^Lys(Ac) (stimulatory) or ^4^Lys(Bu) (inhibitory) could be explained by the ligand-induced selective signaling theory. According to this theory, different GnRH-R agonists may selectively stabilize different receptor conformation and consequently, different signaling pathways may be activated [6,40].

There was a good correlation between the chemorepellent and locomotion decreasing activity of GnRH-III(Dau) and the conjugate with ^4^Lys(Bu), whereas the locomotion enhancer effect of [^4^Lys(Ac)]-GnRH-III(Dau=Aoa) was accompanied with a neutral effect or a slight positive trend in the chemotactic responsiveness of A2058 cells. Our present results are in harmony with studies demonstrating the migration inhibitory effect of GnRH agonists on melanoma [25] and prostate cancer cell lines [54–55]. These studies also suggested that modulation of cell adhesion or actin cytoskeleton remodelling (morphological changes) by GnRH analogs could determine their effects either on vectorial or random cell movements. In spite of the fact that many morphological parameters were examined, our results proved to be modest to demonstrate an unambiguous association between the morphological changes and cell migratory responses of A2058 melanoma cells induced by the conjugates but raised the need to investigate the molecular background of these cell physiological effects. The above-mentioned studies about the antimetastatic activity of different GnRH analogs anticipate that Dau-containing conjugates might influence the (i) expression of cell adhesion molecules (e.g., α3 integrin [25], non-integrin laminin receptor [56]) and (ii) regulate the actin polymerization by interacting with small GTPases (e.g., Rac1, CdC24 [54]) or (iii) interfere with the expression/activity of matrix-degrading enzymes (e.g., MMP-2 [25], urokinase-type plasminogen activator [55]).

Taken together our findings of cell movement and adhesion studies, GnRH-III(Dau=Aoa) and the conjugate containing ^4^Lys(Bu) could have the ability to immobilize the cells at the primary tumor, diminish their spreading and consequently the chance of metastasis development.

## Conclusion

**Figure 8:**
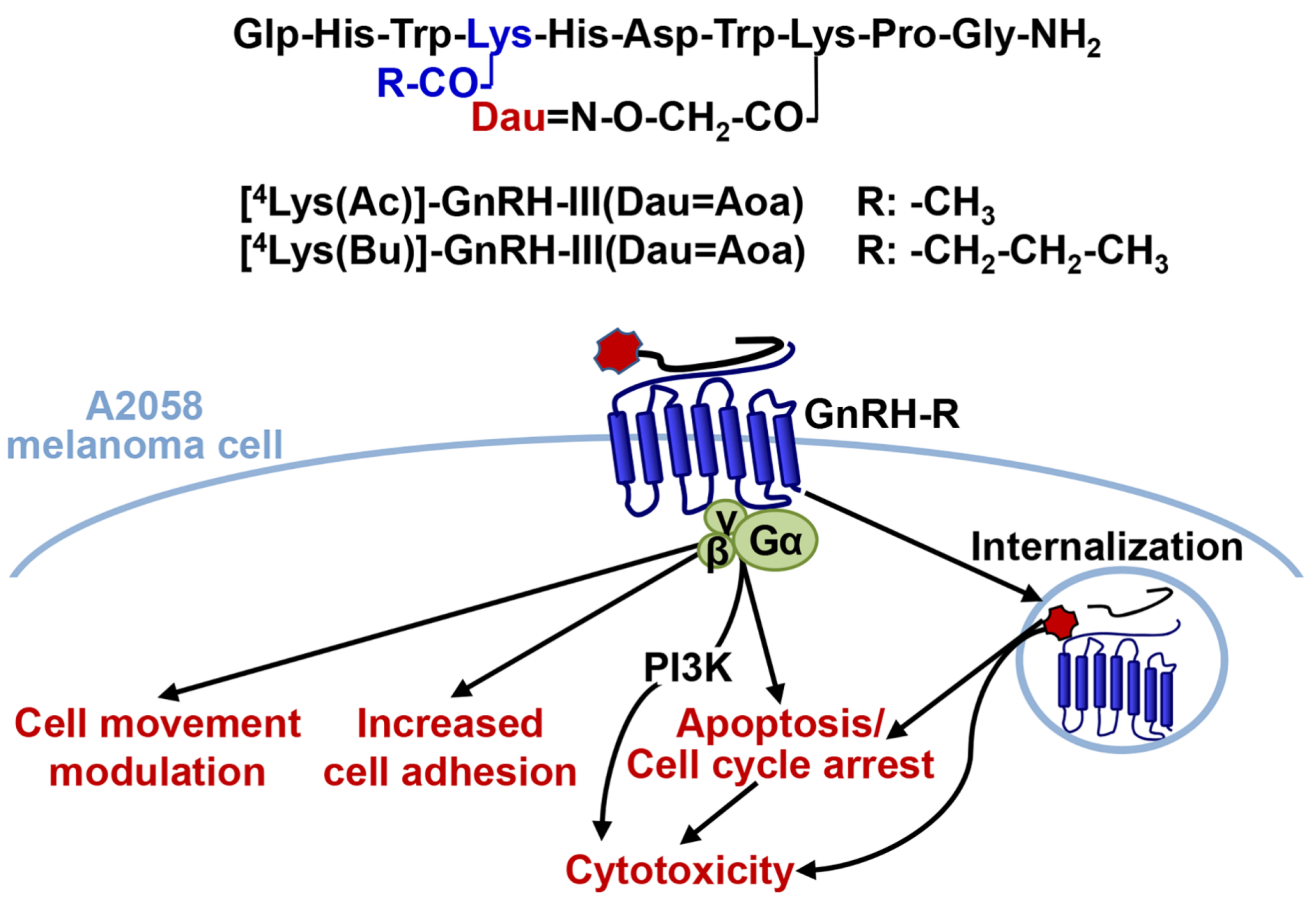
Schematic representation of the proposed mechanisms of the effects of GnRH-III conjugates containing acylated ^4^Lys in A2058 melanoma cell line. Dau: daunorubicin.

In the present study, GnRH-III or its analog substituted with a short-chain fatty acid containing Lys in position 4 was applied as a targeting unit to deliver Dau to melanoma cells. By reading their complex cell physiological activities these conjugates, in which Dau was linked via an oxime bond to ^8^Lys of GnRH-III derivatives, their suitability was demonstrated for targeted melanoma therapy. After the conjugates being internalized by time-dependent manner, they proved to exert an irreversible tumor growth inhibitory effect leading to the conclusion that GnRH-III and its analogs were able to deliver Dau to A2058 human melanoma cells and provide its antineoplastic activity. The presence of short-chain fatty acid containing Lys in position 4 was shown to be accompanied by an increased cellular uptake and a higher long-term cytotoxic activity mediated via a PI3K-dependent signaling (Figure 8). Our findings also suggested that the underlying mechanisms of their antitumor effects, as well as their adhesion modulator and chemotactic/chemokinetic activities depends on the length of the side chain in ^4^Lys. It was clearly shown that [^4^Lys(Bu)]-GnRH-III(Dau=Aoa) possessing a longer, butyryl side chain could reduce the cell viability through its pro-apoptotic effect and the migratory/chemotactic behavior of melanoma cells, as well. Whereas, [^4^Lys(Ac)]-GnRH-III(Dau=Aoa) was appeared to elicit its antitumor effect by arresting the cell cycle in G2/M phase and enhanced the migratory responses of melanoma cells. Our findings indicate the possibility that the locomotory reaction of melanoma cells induced by [^4^Lys(Bu)]-GnRH-III(Dau=Aoa) and GnRH-III(Dau=Aoa) could be associated with the cell adhesion and morphological changes induced by these conjugates.

The present results of measurements on cell adhesion and movement, together with data from the literature [6], suggest that these cell physiological responses could represent a novel therapeutic target of GnRH-III-based conjugates (Figure 8). In addition, we have provided further evidence that the impedimetry and holographic phase imaging are useful and suitable techniques for the characterization of cancer cell behavior and for the evaluation of effects of drug targeting conjugates with small structural differences (e.g., length of the side chain in ^4^Lys).

Based on the overall cell biological effects of [^4^Lys(Bu)]-GnRH-III(Dau=Aoa), the presence of butyrate-containing Lys could provide benefits over the conjugates possessing Lys(Ac) or Ser in position 4. Our results, together with previous data, would suggest the idea that the butyrate could work as a “second drug” in the conjugate. On the basis of the combined cytotoxic, adhesion inducer and cell movement inhibitory effect, [^4^Lys(Bu)]-GnRH-III(Dau=Aoa) proved to be the best candidate in our study for application in the targeted melanoma therapy as a multifunctional antitumor and antimetastatic drug delivery system.

## Supporting Information

File 1 Experimental.

File 2 Analytical parameters and cell biological effects of Dau–GnRH-III conjugates.
