# Supplementary material for: Comparative cell biological study of in vitro antitumor and antimetastatic activity on melanoma cells of GnRH-III-containing conjugates modified with short-chain fatty acids
Source: Beilstein J Org Chem. 2018 Sep 26;14:2495–509. doi: 10.3762/bjoc.14.226 (PMC6178282; doi:10.3762/bjoc.14.226)
Supplement: File 1 — Experimental. [file Beilstein_J_Org_Chem-14-2495-s001.pdf]

Supporting Information File 1

for

Comparative cell biological study of in vitro antitumor and antimetastatic activity on melanoma cells of GnRH-III-containing conjugates modified with short-chain fatty acids

Eszter Lajkó[1], Sarah Spring[1,2], Rózsa Hegedüs[3], Beáta Biri-Kovács[3,4], Sven Ingebrandt[2], Gábor Mező[3,4], László Kőhidai[1]

Address: [1]Department Genetics, Cell- and Immunobiology, Semmelweis University, Nagyvárad tér 4., Budapest, 1089, Hungary, [2]Department of Informatics and Microsystem Technology, University of Applied Sciences Kaiserslautern, Amerikastraße 1, Zweibrücken, 66482, Germany, [3]Research Group of Peptide Chemistry, Hungarian Academy of Sciences, Eötvös Loránd University, Pázmány Péter sétány 1/A, Budapest, 1117, Hungary and [4]Eötvös Loránd University, Faculty of Science, Institute of Chemistry, Pázmány Péter sétány 1/A, Budapest, 1117, Hungary

Email: Eszter Lajkó* - lajesz@gmail.com;

*Corresponding author

Experimental

Table of content

Chemicals S2

Synthesis of GnRH-III-based conjugates S3

Cell culture S5

Western blot analysis S5

Analysis of cellular uptake S6

Cell proliferation and cytotoxicity measurement S6

Inhibition assay for phosphatidylinositol 3-kinase S8

Apoptosis assay S9

Cell cycle analysis S10

Measurement of cell adhesion S11

Chemotaxis assay S11

Morphometry analysis S12

Study of cell movement S13

Statistical analysis S13

References S14

Chemicals

All amino acid derivatives and 4-methylbenzhydrylamine (MBHA) resin were purchased from Reanal (Budapest, Hungary) or NovaBiochem (Läufelfingen, Switzerland). Chemicals for the syntheses (*N*,*N*-Diisopropylethylamine (DIPEA), *N,N'*-diisopropylcarbodiimide (DIC), 1-hydroxybenzotriazole (HOBt), piperidine, 1,8-diazabicyclo[5.4.0]undec-7-ene (DBU), trifluoroacetic acid (TFA), ethanedithiol (EDT), triisopropylsilane (TIS) acetic and butyric anhydride were obtained from Sigma-Aldrich Kft. (Budapest, Hungary), while the solvents (dichloromethane (DCM), *N*,*N*-dimethylformamide (DMF), ethanol and diethyl ether) were

purchased from Reanal or Sigma–Aldrich Kft. Daunorubicin hydrochloride (Dau) was provided to us with a generous gift from IVAX (Budapest, Hungary).

All reagents and solvents were of analytical grade or highest available purity.

Synthesis of GnRH-III-based conjugates

The procedure for the synthesis of GnRH-III-based conjugates used is as described before [1,2]. The GnRH derivatives (GnRH-III(Aoa), [^{4}Lys(Ac)]-GnRH-III(Aoa) and [^{4}Lys(Bu)]-GnRH-III(Aoa) were prepared manually by solid phase peptide synthesis (SPPS) according to Fmoc/tBu chemistry on a Rink-Amide MBHA resin (0.64 mmol/g coupling capacity). Fmoc-Lys(Mtt)-OH was incorporated in position 8 of all three peptides, while Fmoc-Lys(ivDde)-OH in position 4 when Ser was replaced by acylated Lys. The protocol of the synthesis was as follows: (i) DMF washing (4 × 1 min), (ii) Fmoc deprotection with 2% DBU, 2% piperidine in DMF (4 times; 2 + 2 + 5 + 10 min), (iii) DMF washing (8 × 1 min), (iv) coupling of a-Fmoc-protected amino acid derivative: DIC:HOBt (3 equiv each to the resin capacity) in DMF (1 × 60 min), (v) DMF and DCM washing (2-2 × 1 min), (vi) ninhydrin test. After completion of the synthesis of the protected decapeptides, first the ivDde-protecting group of the ε-NH_2 group of ^{4}Lys was removed by 4% hydrazine in DMF (12 × 5 min); afterward, the acylation of the ε-NH_2 group with acetic or butyric anhydride: DIPEA (1:1, mol/mol) for 2 h. After removing the Mtt-protecting group (in case of all three peptides) from the ε-NH_2 group of ^{8}Lys (2% TFA, 2% TIS in DCM for 6 × 5 min), Boc-Aoa-OH was coupled for 2 h using DIC, HOBt coupling reagents (3 equiv each to the amino group). Aminooxyacetylated peptides were cleaved from the resin using a mixture of 95% TFA, 2.5% TIS and 2.5% water (v/v/v) in the presence of 10 equiv free aminooxyacetic acid as “carbonyl capture” reagent for 1.5 h at room temperature and then precipitated with ice-cold diethyl ether, washed three times with diethyl ether and solubilized in 10% acetic acid prior to lyophilization. The crude

products were purified by semipreparative RP-HPLC, analyzed by mass spectrometry and immediately used in the next synthetic step after evaporation of the solvent. The conjugation of daunorubicin (Dau) to the aminooxyacetylated GnRH-III derivatives was carried out in 0.2 M ammonium acetate buffer (pH = 5.0), at a peptide concentration of 10 mg/mL. The reaction mixtures were stirred at room temperature for 16 h and then the bioconjugates were separated by semipreparative RP-HPLC (KNAUER 2501 HPLC system, Knauer GmbH, Bad Homburg, Germany) using a semipreparative Phenomenex Luna C18 column (250 mm × 10 mm) with 10 μm silica (100 Å pore size) (Torrance, CA, USA). Linear gradient elution (0 min 20% B; 5 min 20% B; 50 min 100% B) with eluent A (0.1% TFA in water) and eluent B (0.1% TFA in MeCN-H2O (80:20, v/v)) was used at a flow rate of 4 mL/min. Peaks were detected at 280 nm.

The analytical characterization was similar to the one reported in [1] with some changes. The purified bioconjugates (GnRH-III(Dau=Aoa), [^{4}Lys(Ac)]-GnRH-III(Dau=Aoa) and [^{4}Lys(Bu)]-GnRH-III(Dau=Aoa)), were characterized by analytical RP-HPLC (KNAUER 2501 HPLC system, Knauer GmbH, Bad Homburg, Germany) using either a Hichrom, Vydac 214TP5 C4 (250 × 4.6 mm) column with 5 μm silica (300Å pore size) or a Macherey-Nagel, Nucleosil C18 (250 × 4.6 mm) column with 5 μm silica (100Å pore size) as a stationary phase. Linear gradient elution (0 min 0% B; 5 min 0% B; 50 min 90% B) at a flow rate of 1 mL/min with the same eluents described above. Peaks were detected at 220 nm. Electrospray (ESI)-mass spectrometric analyses were carried out on an Esquire 3000+ ion trap mass spectrometer (Bruker Daltonics, Bremen, Germany). Spectra were acquired in the 50–2500 *m/z* range. Samples were dissolved in a mixture of 50% methanol, 48% water and 2% acetic acid. All the conjugates (GnRH-III(Dau=Aoa), [^{4}Lys(Ac)]-GnRH-III(Dau=Aoa) and [^{4}Lys(Bu)]-GnRH-III(Dau=Aoa)) are water soluble and the dilutions of stock solutions (5×10^{-3} M) for the assays were performed in complete cell culture medium of A2058 cell line.

Cell culture

The effects of Dau-GnRH-III conjugates and the free Dau were investigated in A2058 melanoma cell line showing high metastatic capacity as it was originally derived from a brain metastasis [3]. Cultures of A2058 were maintained at 37 °C in an incubator with a humidified atmosphere of 5% CO_2 and cultured in RPMI 1640 (Sigma-Aldrich, St. Louis, MO, USA) containing 10% FCS (Lonza Group Ltd., Switzerland), L-glutamine (2 mM), 100 µg/ml penicillin/streptomycin (Gibco® / Invitrogen Corporation, New York, NY, USA).

Western blot analysis

To determine the GnRH receptor (GnRH-R) expression of our melanoma model cell, 1.5×10^6 cells/well of A2058 melanoma cells and HT-29 colon carcinoma cells (served as a positive control), were seeded in a 6-well plate. After 24 h culturing, the cells were treated by a TrypLE reagent (Thermo Fisher Scientific, Waltham, MA, USA) composed of recombinant enzyme protecting the surface proteins of the cells and washed with PBS (phosphate-buffered saline, pH = 7.2). The cellular samples were lysed with a buffer containing 25 mM Tris (pH = 7.4), 150 mM NaCl, 1 mM tris(2-carboxyethyl)phosphine , 2 mM EDTA, 1% Triton-X 100 and 1% (v/v) Protease Inhibitor Cocktail (Set III, Calbiochem, San Diego, CA) for 30 min at 4 °C and lysates were centrifuged with 16000 g for 30 min at 4 °C. After the supernatants (protein content: 20 µg/ml, QuBit^{TM} Protein Assay Kit, Thermo Fisher Scientific, Waltham, MA, USA) were mixed with SDS buffer and boiled for 10 min, SDS/PAGE was carried out by using a 10% Tris/Tricine gel and the proteins were blotted on to a PVDF membrane (Bio-Rad Laboratories, Hercules, CA, US). The membranes were incubated with polyclonal anti-GnRH-R antibody (produced in rabbit, dilution: 1:4000, Proteintech Group, Rosemont, IL, USA) for overnight at 4 °C, then with anti-rabbit (dilution: 1:4000, Cell Signaling

Technology, Danvers, MA, USA) secondary antibody, for 1 h at room temperature. The bands were visualized by ECL Western Blot Substrate (Pierce Biotechnology, Rockford, IL, USA).

Analysis of cellular uptake

To study the cellular uptake of conjugates and Dau as the reference drug, A2058 cells (2×10^5 cells/well) were seeded on 12-well plates and cultured for 24 h. The conjugates and Dau were applied in 10^{-5} M final concentration for 1, 4 and 6 h of incubation time. After the treatment, the compounds containing medium were discarded and the cells were removed from the surface with trypsin/EDTA solution (Sigma-Aldrich, St. Louis, MO, USA). After a centrifugation and a washing step with PBS, the cells were resuspended in PBS. The measurements of internalized conjugates are based on the fluorescence activity of Dau. For detecting the intracellular fluorescence intensity of the cells, which is proportional with the cellular uptake, FACSCalibur flow cytometer (Becton Dickinson, San Jose, CA, USA) was used by collecting 10000 cells for each measurement. In case of the negative control, complete cell culture medium was added to the cells instead of conjugates. CellQuest Pro program (Becton-Dickinson, San Jose, CA, USA) was used for the measurement and the evaluation of relative fluorescence intensity (geometric mean channel – GeoMean values).

Cell proliferation and cytotoxicity measurement

The antiproliferative/cytotoxic effect of conjugates compared with Dau was assessed using the xCELLigence SP System (ACEA Biosciences, San Diego, CA, USA). This system uses a so-called E-plate having gold microelectrodes covered the surface of each well and quantifies different cellular activities (e.g. cell proliferation, cell adhesion) by real-time measuring the impedance. The detected impedance change is in direct correlation with the cell number or the electrode surface covered by the attached cells. A relative and dimensionless parameter, Cell index (CI), was calculated from the detected impedance change by the following formula:

$CI = (Z_i - Z_0)/F_i$

where Z_i is the impedance at a given time point, Z_0 is the impedance at $t = 0$ h, and F_i is a constant depending on the frequency ($F_{10kHz} = 15$).

At first, a background measurement was performed by adding 80 µL of complete culture medium and recording the CI for ca 1 h to gain constant baseline impedance curves. The cells were then added to the wells at the concentration of 10^4 cells per well and cultured for 24 h. For the treatment of the cells, the conjugates and the free drug were applied in 10^{-12}–10^{-4} M final concentrations and the cells were monitored for further 72 h.

Identical points of the concentration-course study represent the average of 3 parallel measurements. The proliferation of A2058 cells and the antiproliferative/cytotoxic effects of the tested substances were characterized by a time-course study of Delta CI values. Delta CI refers to the difference of CI value at the time point of cell inoculation and CI value at a given time point. For comparison of the antitumor activity of conjugates and Dau, the IC50 values were calculated from the Delta CI values obtained at 48 and 72 h for each concentration by the integrated software (RTCA 2.0, ACEA Biosciences, San Diego, CA, USA) of xCELLigence System.

In order to evaluate whether the conjugates could elicit their antiproliferative/cytotoxic character in short-term, A2058 cells (10^4 cells/well) were seeded in 96-well plate 24 h before the treatment. The steps of our experiment proceeded in a similar way what was indicated in [4]. After treating the cells with compounds in 10^{-6}–10^{-5} M concentrations, where their most significant cytotoxic effect was detected in case of the long-term treatments, the cells were washed twice with serum-free medium and cultured for further 48 h in serum-containing medium. The viability of cells was determined by alamarBlue-assay (Thermo Fisher Scientific, Waltham, MA, USA) according to the manufacturer's instruction. After 4 h incubation with the alamarBlue® reagent, the fluorescence intensity of the reaction product

was measured with LS-50B Luminescence Spectrometer (Perkin Elmer Ltd., Buckinghamshire United Kingdom) by using λ = 560 nm for excitation and λ = 590 nm for detection of the emitted light.

Each data represents the mathematical average of six parallels. The decrease in the fluorescence intensity caused by the different treatments was normalized to the identical control and this value was given as ‘Viability’ in percent.

Both in long- and short-term measurements, the wells loaded with an identical volume of fresh cell culture medium were served as control.

Inhibition assay for phosphatidylinositol 3-kinase

The role of phosphatidylinositol 3-kinase (PI3K) in the antiproliferative/cytotoxic effects of conjugates was studied by commonly used inhibitors – wortmannin and LY294002 (Sigma-Aldrich, St. Louis, MO, USA) – of the enzyme.

A2058 cells were prepared in 96-well plates as it was aforementioned in ‘Cell proliferation and cytotoxicity measurement’ section. After 24h culturing, the cells were pretreated with wortmannin (50 nM), LY294002 (15 μM) or with an adequate volume of DMSO, their solvent, for 30 min. The conjugates and Dau in 10^{-6} and 10^{-5} M final concentrations were additionally loaded to the wells and the cells were further incubated for 48 h. The antiproliferative/cytotoxic effect of conjugates was determined in the following combinations: cells pretreated with DMSO were added with the control medium (C_c) or a compound (G_c); cells pretreated with wortmannin or LY294002 were assayed for the control medium (C_{inh}) or a compound (G_{inh}). The number of viable cells was determined by the alamarBlue-assay as it was described in ‘Cell proliferation and cytotoxicity measurement’ chapter. To analyze the effects of inhibitors on the actions of conjugates, the inhibition index was calculated by

normalizing the viability of conjugate-treated cells with that of the control cells, and these indices of groups pretreated with inhibitors or DMSO were statistically compared.

Apoptosis assay

The apoptotic effect of conjugates and Dau was determined by a FACSCalibur flow cytometer (Becton Dickinson, San Jose, CA, USA) after staining with annexin V-FITC (Sony Biotechnology, Weybridge, UK) and NucleoCounter® NC-250™ cell analyzer (ChemoMetec A/S, Lillerød, Denmark) using Vitabright-48™, a cell-permeable fluorescent dye (ChemoMetec A/S, Lillerød, Denmark).

The sample preparation for the apoptosis measurements and the flow cytometric analysis bears a close resemblance to the description in [4]. Prior to the treatment with the compounds, the cells were seeded on 12-well plates in 2×10^5 cells/well density and cultured for 24 h. The compounds were added to the cells in 10^{-5} M concentration and the incubation time was 24 h. The cells were dissociated by a TrypLE reagent (Thermo Fisher Scientific, Waltham, MA, USA) composed of recombinant enzyme protecting the cell's surface proteins and it was followed by a centrifugation and a washing step with PBS. For the Annexin V-staining, the annexin binding buffer (Becton Dickinson, San Jose, CA, USA) was added to resuspend the cells. Before labeling with annexin V-FITC, the cells were measured by a flow cytometer for controlling the autofluorescence of cells in case of each treatment group. Then, the cells were labeled with annexin V-FITC for 15 min in dark and analyzed by a flow cytometer using 10000 cells for each measurement.

For the measurement and the data analysis, CellQuest Pro software (Becton Dickinson, San Jose, CA, USA) was used. For the numerical comparison, the detected values of the annexin V-labeled, viable cells were adjusted with non-labeled samples and the percentage of annexin

positive cells in the treated groups was compared to the percentage of annexin positive control cells.

NucleoCounter® NC-250™, an image cytometer, was also used to detect the apoptosis by quantifying the fluorescence intensity of Vitabright-48™ reacting with the free intracellular thiols (e.g. reduced glutathione). After the staining step, the cells were immediately loaded into an NC-Slide and analyzed using the NucleoCounter® NC-250™ system. The decrease in the thiol level (in the relative fluorescence intensity of Vitabright-48™) is correlated with the apoptosis. By comparing fluorescence intensity of treated cells to that of the controls, the fraction of apoptotic cells (cells with low thiol level) can be determined and given as percentage by using the software of this equipment.

Cell cycle analysis

To study whether the conjugates and Dau exhibit their antitumor effect by interfering with the cell cycle progression of A2058 cells, FACSCalibur flow cytometer (Becton Dickinson, San Jose, CA, USA) was used to measure the DNA content of cells with propidium iodide (Sigma-Aldrich, St. Louis, MO, USA) intercalated stoichiometrically to the double-stranded DNA.

A2058 cells were seeded, treated and harvested in the same way described in section 'Apoptosis assay'; then, the cells were fixed in ice-cold 70% ethanol and kept at −20 °C for 24 h. The samples were centrifuged and resuspended in RNase (100 μg/ml, Sigma-Aldrich, St. Louis, MO, USA) containing citric acid/sodium phosphate buffer (pH = 7.8). Propidium iodide was added to the sample in 10 μg/ml concentration right before the flow cytometric measurements collecting at 25000 cells/sample.

Data were analyzed by CellQuest Pro and Flowing 2.5.1 (Turku Centre of Biotechnology, Turku, Finland) software. For aggregate/debris discrimination, FL2-Width vs FL2-Area plot

was used and the gated cells were displayed in FL2-Area histogram to assign percentage values to each population of cell cycle stages.

Measurement of cell adhesion

The effect of Dau-GnRH-III conjugates on cell adhesion of model cell was assessed using the xCELLigence SP System (ACEA Biosciences, San Diego, CA, USA).

The adhesion assay, analogous to the description in [5], was started with a background measurement as mentioned above in the section “Cell proliferation and cytotoxicity measurement”. In the next steps, the compounds were added in the concentration range of 10^{-8}–10^{-5} M and the cells were loaded into the wells at a concentration of 10^4 cells/well. The adhesion of A2058 cells was monitored every 20 sec for at least 12 h at 10 kHz.

The attachment and spreading of model cell were characterized by a time-course study of the Delta CI values. The Delta CI values were calculated by the integrated software (RTCA 2.0). The Delta CI values obtained at 3 h of incubation for each concentration of conjugates were normalized to the untreated control and were given as a percentage. Each data represents the mathematical average of three parallels.

Chemotaxis assay

Chemotactic responsiveness of A2058 cell lines was measured as previously reported [4] in a NeuroProbe® MBB 96 chamber (NeuroProbe, Gaithersburg, MD, USA) by using polycarbonate filters with pore size 8 μm. To quantify the number of the chemotactically active cells, the alamarBlue-assay was applied as explained above. The ‘Chemotaxis index (Chtx. ind.) plotted in Figure 6 was calculated from the average of 8 parallel measurements and expressed as a percentage of control.

Morphometry analysis

The experimental set up we used for the morphometry analysis can be also found in [6]. The Holomonitor^{TM} M4 microscope (Phase Holographic Imaging AB, Lund, Sweden) was used for imaging and tracking the morphological changes of A2058 cells induced by the conjugates and Dau. This technique is dedicated to analyzing the morphology and movement of adherent cells by recording 3D, holographic phase contrast images and tracking them over a given time period. The principle behind this method is the detection of the phase shift of the probing laser light transmitting or reflecting through a cell sample and comparison to the reference light [7,8].

A2058 cells were seeded (2.5×10^5 cells) in a petri dish (diameter: 35 mm) and allowed to adhere for 24 h. After automatic calibration of the background and microscope objective (20×), one field of each sample was focused on by the digital autofocus feature. The cells were then treated with the test compounds at the final concentrations of 10^{-7}–10^{-5} mol/L, or with an equivalent volume of culture medium, and captured every 30 sec for 2 h.

For the evaluation, ca. 50 cells per time-lapse sequence were identified and selected by the minimum error histogram-based threshold algorithm of the HStudio^{TM} M4 2.7.1 software (Phase Holographic Imaging AB, Lund, Sweden). The morphological changes were automatically analyzed over time by selecting and tracking individual cells.

For the morphometry analysis, different basic and complex morphological parameters were displayed. The (i) area (surface area of the image occupied by a cell) and (ii) thickness (average spatial cell thickness) were grouped as basic metrics, while the more complex parameters relating to cell shape were: (iii) the eccentricity (how elongated a cell is comparing to a circle), (iv) the hull convexity (how different the 3D shape of a cell is compared to a perfect convex shape) and (v) the irregularity (how different the circumference of a cell is compared to the circumference of a perfect circle).

Study of cell movement

The movement A2058 cells induced by the conjugates and Dau (their chemokinetic effect) was investigated by Holomonitor^{TM} M4 microscope. Once the morphological changes of A2058 cells have been tracked, their cell movement was also be followed and registered over time. For the characterization of the chemokinetic activity of the conjugates, the following parameters were quantified: (i) migration (shortest distance between the starting point and the end point); (ii) motility (actual distance travelled by a cell from the starting point to the end point) and (iii) motility speed (ratio of actual path to time). For the evaluation of the time-lapse recordings, the same settings were used that in case of the morphometry study.

Statistical analysis

Data obtained from each experiment represent mathematical averages and ±SD values. Evaluation of the results was performed by using the MS Excel, OriginPro 2016 (OriginLab Corporation, Northampton, MA, USA). Statistical analysis was done by application of ANOVA using OriginPro 2016. Tukey's post hoc test was used to assess the statistical significance of means. The levels of significance are shown as follows: *: $p < 0.05$; **: $p < 0.01$; ***: $p < 0.001$.

References

1. Hegedus, R.; Manea, M.; Orban, E.; Szabo, I.; Kiss, E.; Sipos, E.; Halmos, G.; Mezo, G. *Eur. J. Med. Chem.* **2012,** *56*, 155-165. doi:10.1016/j.ejmech.2012.08.014
2. Manea, M.; Leurs, U.; Orbán, E.; Baranyai, Z.; Ohlschlager, P.; Marquardt, A.; Schulcz, A.; Tejeda, M.; Kapuvári, B.; Továri, J.; Mező, G. *Bioconjug. Chem.* **2011,** *22*, 1320-1329. doi:10.1021/bc100547p
3. Liotta, L. A.; Mandler, R.; Murano, G.; Katz, D. A.; Gordon, R. K.; Chiang, P. K.; Schiffmann, E. *Proc. Natl. Acad. Sci. U S A* **1986,** *83*, 3302-3306. doi:10.1073/pnas.83.10.3302
4. Lajko, E.; Banyai, P.; Zambo, Z.; Kursinszki, L.; Szoke, E.; Kohidai, L. *Cancer Cell Int.* **2015,** *15*, 119. doi:10.1186/s12935-015-0271-4
5. Lajko, E.; Szabo, I.; Andody, K.; Pungor, A.; Mezo, G.; Kohidai, L. *J. Pept. Sci.* **2013,** *19*, 46-58. doi:10.1002/psc.2472
6. Lajko, E.; Tuka, B.; Fülöp, F.; Krizbai, I.; Toldi, J.; Magyar, K.; Vécsei, L.; Kőhidai, L. *J. Neural Transm.* **2018,** *125*, 899-912. doi:10.1007/s00702-018-1839-7
7. Persson, J.; Mölder, A.; Pettersson, S.; Alm, K., Cell motility studies using digital holographic microscopy. In *Microscopy: Science, Technology, Applications and Education*, Vilas, M.; Díaz, J., Eds.; Formatex Research Center: Badajoz, Spain, 2010; pp 1063-1072.
8. Peter, B.; Nador, J.; Juhasz, K.; Dobos, A.; Korosi, L.; Szekacs, I.; Patko, D.; Horvath, R. *J. Biomed. Opt.* **2015,** *20*, 067002. doi:10.1117/1.jbo.20.6.067002
