# Supplementary material for: Comparative cell biological study of in vitro antitumor and antimetastatic activity on melanoma cells of GnRH-III-containing conjugates modified with short-chain fatty acids
Source: Beilstein J Org Chem. 2018 Sep 26;14:2495–509. doi: 10.3762/bjoc.14.226 (PMC6178282; doi:10.3762/bjoc.14.226)
Supplement: File 2 — Analytical parameters and cell biological effects of Dau–GnRH-III conjugates. [file Beilstein_J_Org_Chem-14-2495-s002.pdf]

Supporting Information File 2

for

Comparative cell biological study of in vitro antitumor and antimetastatic activity on melanoma cells of GnRH-III-containing conjugates modified with short-chain fatty acids

Eszter Lajkó[1], Sarah Spring[1,2], Rózsa Hegedüs[3], Beáta Biri-Kovács[3,4], Sven Ingebrandt[2], Gábor Mező[3,4], László Kőhidai[1]

Address: [1]Department Genetics, Cell- and Immunobiology, Semmelweis University, Nagyvárad tér 4., Budapest, 1089, Hungary, [2] Department of Informatics and Microsystem Technology, University of Applied Sciences Kaiserslautern, Amerikastraße 1, Zweibrücken, 66482, Germany, [3]Research Group of Peptide Chemistry, Hungarian Academy of Sciences, Eötvös Loránd University, Pázmány Péter sétány 1/A, Budapest, 1117, Hungary and [4]Eötvös Loránd University, Faculty of Science, Institute of Chemistry, Pázmány Péter sétány 1/A, Budapest, 1117, Hungary

Email: Eszter Lajkó* - lajesz@gmail.com;

*Corresponding author

Analytical parameters and cell biological effects of Dau-GnRH-III conjugates

Table of content

Figure S1: S2

Figure S2: S2

Figure S3: S2

Figure S4: S3

Figure S5: S4

Table S1: S5

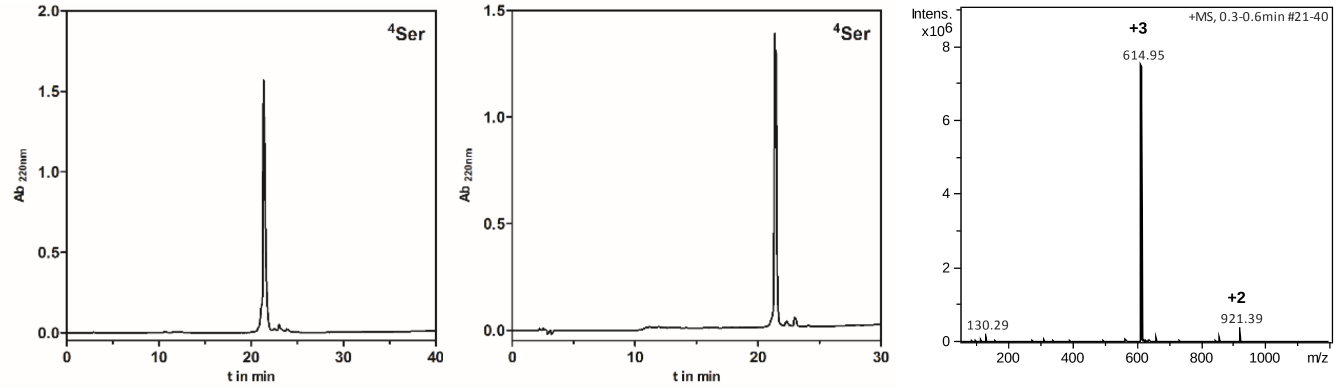

Figure S1: RP-HPLC profile (left: C4 column, centre: C18 column) and ESI-ion trap mass spectrum (right) of GnRH-III(Dau=Aoa). (MW_{cal}/MW_{exp} = 1841.89/1841.85 g/mol, *fragment ion: amino sugar loss of Dau).

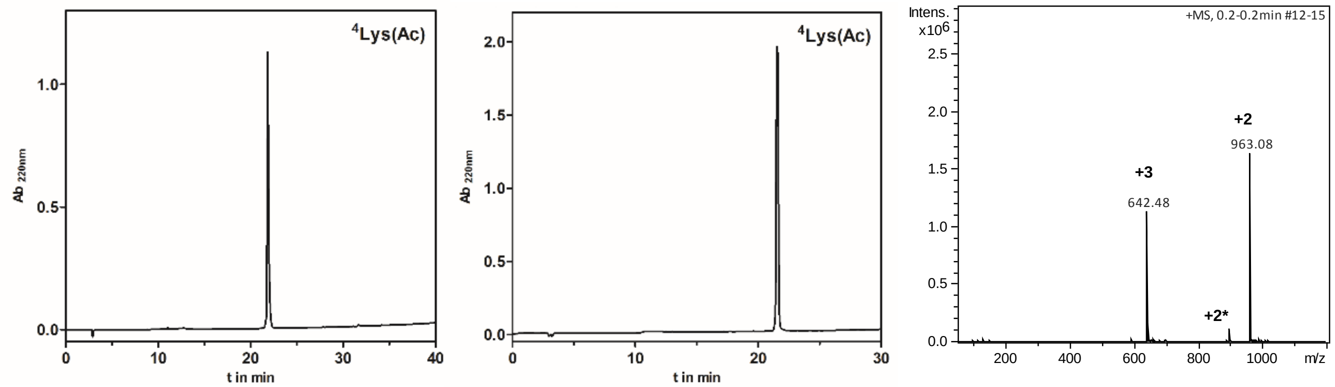

Figure S2: RP-HPLC profile (left: C4 column, centre: C18 column) and ESI-ion trap mass spectrum (right) of [4Lys(Ac)]-GnRH-III(Dau=Aoa). (MW_{cal} /MW_{exp} = 1925.02/1924.14 g/mol).

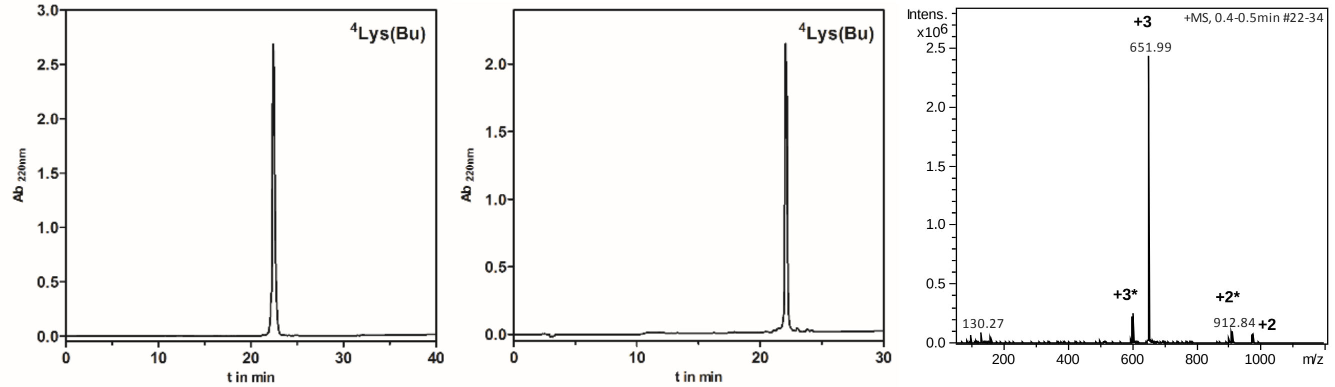

Figure S3: RP-HPLC profile (left: C4 column, centre: C18 column) and ESI-ion trap mass spectrum (right) of [4Lys(Bu)]-GnRH-III(Dau=Aoa). (MW_{cal} /MW_{exp} = 1953.07/1952.97 g/mol).

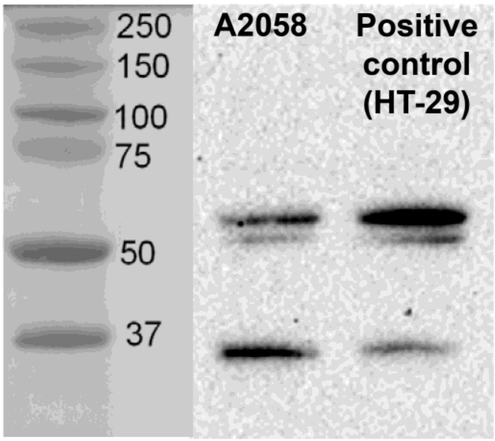

Figure S4: Detection of GnRH receptor in A2058 melanoma cells by Western blotting.

A representative blot of two independent experiments is shown. The bands of the molecular weight marker represent a mixture of recombinant proteins of precise molecular weights (kD).

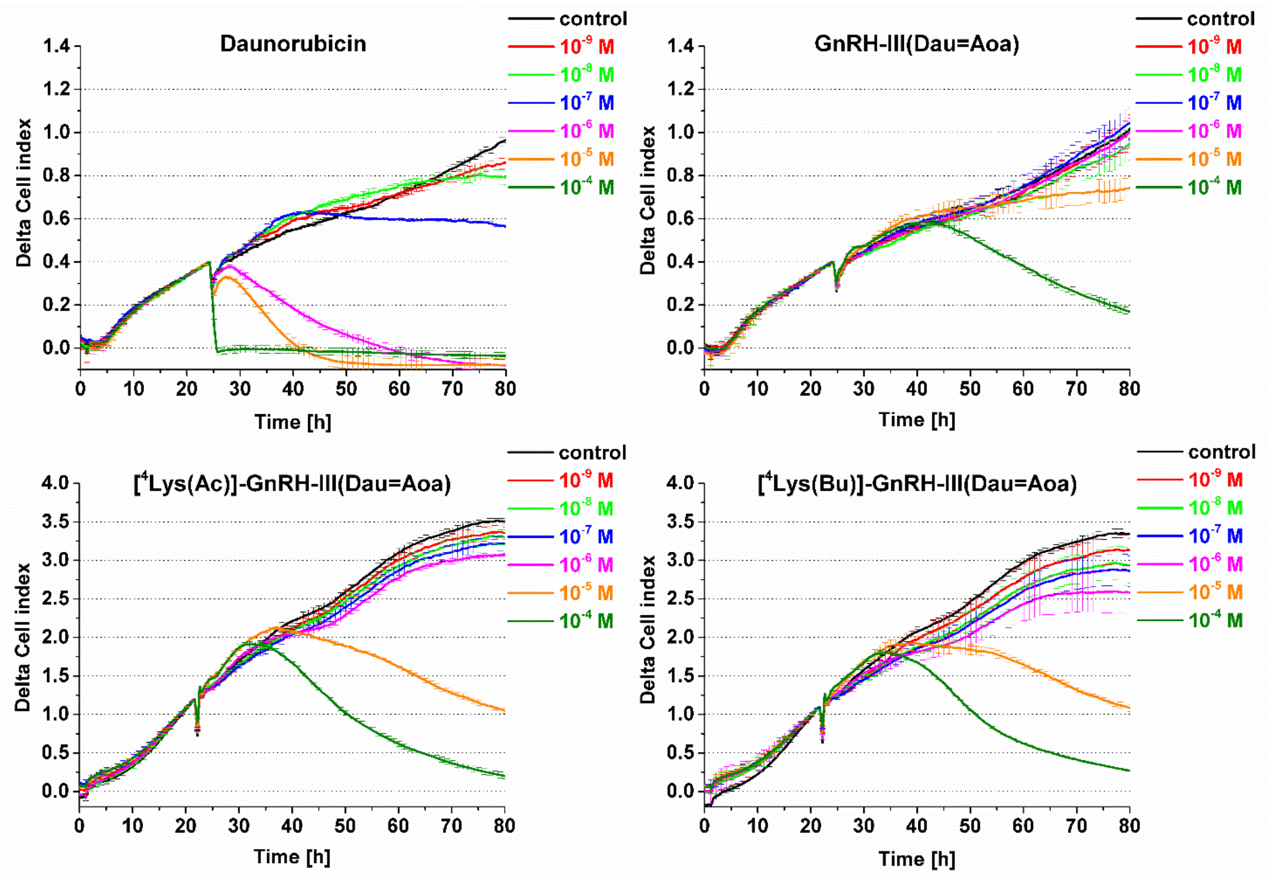

Figure S5: Time course study of the effect of the conjugates and daunorubicin (Dau) on A2058 cell viability by xCELLigence system

The Delta Cell index refers to the difference of the Cell index value at the point in time of cell inoculation and the cell index value at another given point in time.

Data shown in the figures represent mathematical averages of three parallels and ±SD values.

Table S1: Morphological changes of A2058 melanoma cells induced by the conjugates

Compounds/ concentrations	Area [%]	Thickness [%]	Eccentricity [%]	Hull convexity [%]	Irregularity [%]
**Daunorubicin (Dau)**					
10^{-7} M	94.5 ± 3.72	101.8 ± 1.95	95.2 ± 2.25	103.4 ± 0.29	94.5 ± 2.41
10^{-6} M	124.1*** ± 4.64	95.4 ± 1.87	96.6 ± 1.83	100.1 ± 0.23	109.9* ± 2.02
10^{-5} M	99.1 ± 4.1	104.5 ± 2.12	104.4 ± 1.84	101.2 ± 0.29	102.6 ± 2.54
**GnRH-III(Dau=Aoa)**					
10^{-7} M	115.7* ± 4.67	94.9 ± 2.42	103.8 ± 2.33	95.8 ± 0.18	103.1 ± 3.26
10^{-6} M	104.4 ± 3.45	85.6*** ± 1.45	107.4* ± 1.87	94.3 ± 0.23	110.9* ± 2.43
10^{-5} M	112.6* ± 4.31	91.2** ± 1.31	103.6 ± 2.07	95.6 ± 0.25	110.2* ± 2.1
**[^{4}Lys(Ac)]-GnRH-III(Dau=Aoa)**					
10^{-7} M	84.2* ± 3.32	102.1 ± 1.84	93.2 ± 1.4	101.4 ± 0.19	92 ± 2.23
10^{-6} M	92.5 ± 3.5	95.7 ± 1.94	98.5 ± 1.78	99.2 ± 0.24	95.2 ± 2.36
10^{-5} M	89.7 ± 3.66	103.1 ± 2.21	93.8 ± 2.08	99.2 ± 0.34	81** ± 1.99
**[^{4}Lys(Bu)]-GnRH-III(Dau=Aoa)**					
10^{-7} M	100.4 ± 5.23	99.9 ± 1.65	106.5 ± 2.4	103.1 ± 0.13	97.8 ± 3.11
10^{-6} M	114* ± 5.64	85.7** ± 1.85	95.2 ± 3.13	96.6 ± 0.25	117.8** ± 2.99
10^{-5} M	97.2 ± 5.19	91.9** ± 1.84	100.1 ± 2.15	101 ± 0.19	103.1 ± 2.9

Area (μm^2) – surface area of the image that is occupied by the cell; Thickness (μm) – average thickness of the cell; Eccentricity – how elongated a cell comparing to a circle shape; Hull convexity – how different the 3D shape of a cell from the perfect convex shape; Irregularity – how different the circumference of a cell from the circumference of a perfect circle

Data represent averages and ±SD values calculated for 50 cells/group in 180 consecutive frames.

The presented data were calculated by HoloStudio™ M4 2.5 and analyzed by Origin Pro 2016. The levels of significance are shown as follows: *: $p<0.05$; **: $p<0.01$; ***: $p<0.001$
